# An atlas of trait associations with resting-state and task-evoked human brain functional organizations in the UK Biobank

**DOI:** 10.1162/imag_a_00015

**Published:** 2023-09-07

**Authors:** Bingxin Zhao, Tengfei Li, Yujue Li, Zirui Fan, Di Xiong, Xifeng Wang, Mufeng Gao, Stephen M. Smith, Hongtu Zhu

**Affiliations:** Department of Statistics and Data Science, University of Pennsylvania, Philadelphia, Pennsylvania, USA; Department of Statistics, Purdue University, West Lafayette, Indiana, USA; Department of Radiology, University of North Carolina at Chapel Hill, Chapel Hill, North Carolina, USA; Biomedical Research Imaging Center, School of Medicine, University of North Carolina at Chapel Hill, Chapel Hill, North Carolina, USA; Department of Biostatistics, University of North Carolina at Chapel Hill, Chapel Hill, North Carolina, USA; Wellcome Centre for Integrative Neuroimaging, FMRIB, Nuffield Department of Clinical Neurosciences, University of Oxford, Oxford, United Kingdom; Department of Genetics, University of North Carolina at Chapel Hill, Chapel Hill, North Carolina, USA; Department of Computer Science, University of North Carolina at Chapel Hill, Chapel Hill, North Carolina, USA; Department of Statistics and Operations Research, University of North Carolina at Chapel Hill, Chapel Hill, North Carolina, USA

**Keywords:** brain function, functional connectivity, human traits, mental Health, resting fMRI, task fMRI, UK Biobank

## Abstract

Functional magnetic resonance imaging (fMRI) has been widely used to identify brain regions linked to critical functions, such as language and vision, and to detect tumors, strokes, brain injuries, and diseases. It is now known that large sample sizes are necessary for fMRI studies to detect small effect sizes and produce reproducible results. Here, we report a systematic association analysis of 647 traits with imaging features extracted from resting-state and task-evoked fMRI data of more than 40,000 UK Biobank participants. We used a parcellation-based approach to generate 64,620 functional connectivity measures to reveal fine-grained details about cerebral cortex functional organizations. The difference between functional organizations at rest and during task was examined, and we have prioritized important brain regions and networks associated with a variety of human traits and clinical outcomes. For example, depression was most strongly associated with decreased connectivity in the somatomotor network. We have made our results publicly available and developed a browser framework to facilitate the exploration of brain function-trait association results (http://fmriatlas.org/).

## 1 Introduction

Functional magnetic resonance imaging (fMRI) is a noninvasive and comprehensive method of assessing functional organizations of the human brain. By measuring blood oxygen level dependent (BOLD) signal changes, fMRI can map complex brain functions and estimate neural correlations between different brain regions (Power et al., 2011). When the subject is performing a specific task, fMRI can detect brain signals and regions that link to the task (Ogawa et al., 1990), which is known as task-evoked fMRI. As an alternative, resting-state fMRI can observe brain signals during rest and measure intrinsic functional organization without performing any tasks (Biswal et al., 1995). Both task-evoked and resting-state fMRIs have been widely used in clinical and epidemiological neuroscience research to explore the relationship between inter-individual variations in brain function and human traits. For example, resting-state functional abnormalities are frequently observed in neurological and psychiatric disorders, such as Alzheimer's disease (Agosta et al., 2012), attention-deficit/hyperactivity disorder (ADHD) (Posner et al., 2014), schizophrenia (Hu et al., 2017), and major depressive disorder (MDD) (Mulders et al., 2015). fMRI has also been used to identify the influence of multi-system diseases and complex traits, such as diabetes (Macpherson et al., 2017), alcohol consumption (Ewing et al., 2014), and dietary behaviors (Zhao et al., 2017), on brain functions.

A major limitation of most fMRI association studies has been their small sample size, which is usually less than one hundred or a few hundreds. As functional connectivity measures may be noisy and have large intra-subject variations (Elliott et al., 2020), it may be difficult to replicate fMRI-trait associations found in small studies (Marek et al., 2022). This problem can be resolved statistically by increasing the sample size of fMRI studies, which can detect weaker signals and reduce the uncertainty of the results. For example, Marek et al. (2022) showed that when the sample size is larger than 2,000, brain-behavioral phenotype associations can become more reproducible. However, the high assessment costs of fMRI may make it difficult to increase sample sizes sufficiently to collect the necessary data in every study. In the last few years, several large-scale fMRI datasets involving over 10,000 subjects have become publicly available, including the Adolescent Brain Cognitive Development (Chaarani et al., 2021) (ABCD), the Chinese Imaging Genetics (CHIMGEN) (Xu et al., 2020), and the UK Biobank (Miller et al., 2016) (UKB). Particularly, the UKB study collected a rich variety of human traits and disease variables (Bycroft et al., 2018), providing the opportunity to discover and validate fMRI-trait associations in a large-scale cohort.

**Fig. 1.**
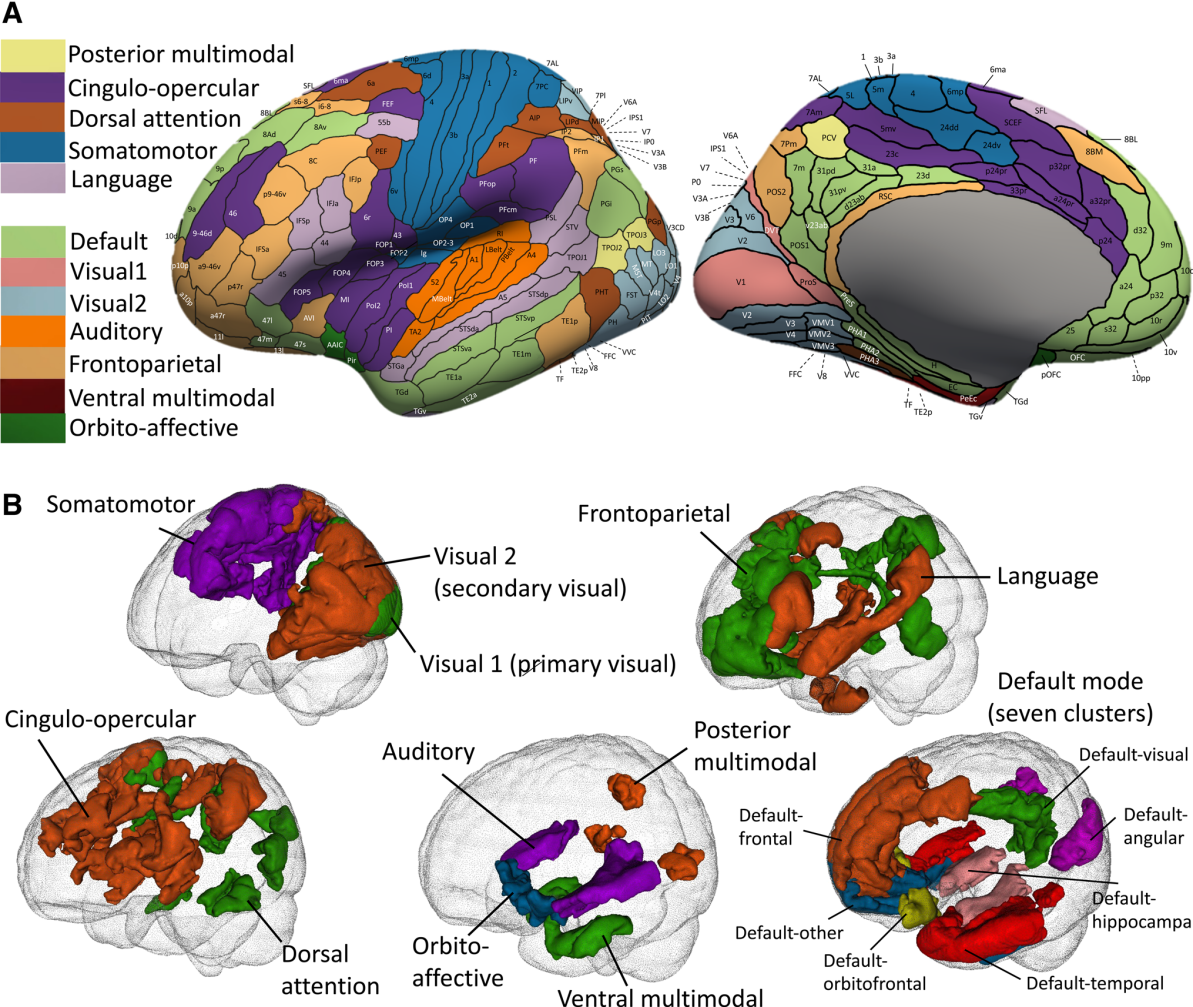
Illustration of functional areas and networks in the Glasser360 atlas. (A) Functional areas defined in the Glasser360 atlas (left hemisphere). See Table S1 for information on these areas and Figure S1 for maps of the whole brain (both hemispheres). Visual1, the primary visual network; Visual2, the secondary visual network. (B) Annotation of the 12 functional networks in the human brain. The default mode network (bottom right) is further divided into seven clusters, mainly based on their physical locations.

Based on fMRI data from more than 40,000 subjects in the UKB study, we investigated resting-state and task-evoked functional organizations and their associations with human traits and health outcomes. By processing raw fMRI images from the UKB study, we represented the brain as a functional network containing 360 brain areas in a parcellation (Glasser et al., 2016) developed using the Human Connectome Project (Van Essen et al., 2013) (HCP) data (referred to as the Glasser360 atlas, Fig. 1, Fig. S1, and Table S1). The Glasser360 atlas contained 64,620 (360 × 359/2) full correlation measures to represent the functional connections among 360 brain areas in 12 functional networks (Ji et al., 2019): the primary visual, secondary visual, auditory, somatomotor, cingulo-opercular, default mode, dorsal attention, frontoparietal, language, posterior multimodal, ventral multimodal, and orbito-affective networks. Compared to the functional connectome data provided by the UKB study, which were generated from whole brain spatial independent component analysis (ICA) (Alfaro-Almagro et al., 2018; Beckmann & Smith, 2004; Hyvarinen, 1999), the parcellation-based approach (like Glasser360) can provide more fine-grained details of brain functional organizations.

We explored brain-trait associations by performing a systematic analysis with 647 traits and diseases (selected to represent a wide range of traits and health conditions) using a discovery-validation design. Functional brain regions and networks were found to be strongly associated with a range of disorders and complex traits. In order to evaluate how the choice of parcellation may impact our results, we additionally applied another parcellation (Schaefer et al., 2018) on the same datasets, which divided the brain into 200 regions, referred to as the Schaefer200 atlas (Fig. S2 and Table S2). We found that the two parcellations can yield similar conclusions and patterns, whereas the Glasser360 atlas can provide more biological insights due to its finer partitioning. We also explored the differences between resting-state and task-evoked functional organizations, as well as age- and sex-related effects. Numerous studies have investigated the impacts of age and sex disparities on brain structures and functions. However, the specific locations and patterns of these identified differences can vary across studies (Ritchie et al., 2018; Scheinost et al., 2015). By leveraging parcellation-based data from the comprehensive UKB study, our aim is to provide a more in-depth exploration of differences in resting-state functional connectivity and their correlations with age and sex.

In order to facilitate the exploration of our extensive results obtained from large-scale fMRI data, we have developed an interactive browser tool, accessible at http://fmriatlas.org/. This tool acts as a gateway for users to navigate and delve deeper into our research findings. While we will highlight several pivotal discoveries in the forthcoming sections of the main body, we urge readers to consult the Supplementary Materials and utilize our online tool for a more comprehensive understanding and discovery of additional patterns. It is worth noting that our bioinformatics resource will be regularly updated and broadened to include new findings and data. Future updates will encompass integration with new brain parcellations, alternative data processing pipelines, and the addition of future large-scale fMRI datasets. These improvements will further augment the tool's functionality, keeping it current and providing the research community with a continually updated platform for the exploration of fMRI data.

## 2 Materials and Methods

### 2.1 Brain imaging data

We generated functional connectivity measures from the raw resting and task fMRI data downloaded from the UKB data categories 111 and 106, respectively. Details of image acquisition and preprocessing procedures were summarized in the Supplementary Note. We mapped the preprocessed images onto the Glasser360 atlas (Glasser et al., 2016), which projected the fMRI data onto a brain parcellation with 360 areas, resulting in a 360 × 360 functional full correlation matrix for each subject (full correlation). The Glasser360 atlas was originally a surface-based parcellation (Dickie et al., 2019), and has been converted into a volumetric atlas that is compatible with UKB data. The 360 brain functional areas were grouped into 12 functional networks (Ji et al., 2019), including the primary visual, secondary visual, auditory, somatomotor, cingulo-opercular, default mode, dorsal attention, frontoparietal, language, posterior multimodal, ventral multimodal, and orbito-affective (Table S1). The 64,620 (360 × 359/2) functional connectivity measures were studied in our main analyses. These high-resolution fMRI traits provided fine details on cerebral cortex functional organization and allowed us to compare the resting and task-evoked functional organizations.

To investigate the potential cross-parcellation variability, we also projected the fMRI data onto the Schaefer200 atlas (Schaefer et al., 2018) and obtained the 200 × 200 functional connectivity matrices (full correlation, Table S2). The resting and task fMRI data from the HCP study were also used in our analysis. In addition to functional connectivity measures, we generated amplitude measures for the brain functional areas in the Glasser360 atlas, which quantified the brain functional activity (Alfaro-Almagro et al., 2018; Bijsterbosch et al., 2017; Zou et al., 2008). Precise mathematical definitions and previous examples of amplitude applications in UKB and HCP studies can be found in Bijsterbosch et al. (2017).

### 2.2 Consistency, reliability, and comparison of resting and task fMRI

Following the previous Glasser360 paper (Glasser et al., 2016), we first checked the group mean maps of two independent sets of UKB subjects (UKB phases 1 & 2 data and UKB phase 3 data). In the UKB phase 3 data, we removed the relatives of early phase subjects. We obtained the group means for each functional connectivity measure separately in the two datasets. To measure the similarity/consistency of the two sets of group means, we calculated their Pearson correlation. For both the resting and task fMRI, the same analysis was conducted, and we also compared the group mean maps between resting and task fMRI by using Pearson correlation. Next, we evaluated the intra-subject reliability by using repeated images. We generated and compared the group mean maps for the original visit and repeated visit separately as we did in the above two-phase analysis. For each functional connectivity measure, we also checked the individual-level differences by taking the Pearson correlation across all subjects with two visits. Finally, we repeated the group mean and intra-subject reliability analyses by using repeated scans in the HCP study.

### 2.3 Age effects and sex differences analysis

Between 2006 and 2010, approximately half a million participants aged 40 to 69 were recruited for the UKB study. The UKB imaging study is an ongoing project to re-invite 100,000 UKB participants to collect multi-modal brain and body imaging data (Littlejohns et al., 2020). We used the UKB phases 1 to 4 data (released up through early 2021, *n *= 40,880 for resting fMRI and 34,671 for task fMRI) in our analysis. The age (at imaging) range of subjects was 44 to 82 (mean age = 64.15, standard error = 7.74), and the proportion of females was 51.6%. In the age and sex analysis, we fitted the following model for each fMRI trait: y=x+z+xzα+wη+∈, where y is the standardized fMRI trait, x is the standardized age, z is the sex factor (0 for female and 1 for male), w is the set of adjusted covariates, $\beta_{1}$ is the main effect of x on y, $\beta_{2}$ is the main effect of z on y, α is the effect of age-sex interaction term xz on y, η represents effects of covariates, and ∈ is the random error variable. We adjusted the following covariates: imaging site, head motion, head motion-squared, brain position, brain position-squared, volumetric scaling, height, weight, body mass index, heel bone mineral density, and the top 10 genetic principal components. For each continuous trait or covariate variable, we removed values greater than five times the median absolute deviation from the median. These removed values will be treated as missing entries in the dataset. We performed the analysis in a discovery-validation design and only reported the results that were significant in both discovery and validation datasets (at different significance levels). Specifically, as in previous studies (Zhao et al., 2022), we used the UKB white British subjects in phases 1 to 3 data (*n *= 33,795 for resting and 28,907 for task) as our discovery sample. The assignment of ancestry in UKB was based on self-reported ethnicity and has been verified in Bycroft et al. (2018). The UKB non-British subjects in phases 1 to 3 data and the individuals in newly released UKB phase 4 data (*n *= 5,961 for resting and 4,884 for task, removed relatives of the discovery sample) were treated as the validation sample. We reported *P* values from the two-sided t test and focused on the results that were significant at the Bonferroni significance level (7.73 × 10^-7^, 0.05/64,620 for the Glasser360 atlas; and 2.51 × 10^-6^, 0.05/19,900 for the Schaefer200 atlas) in the discovery dataset and were also significant at nominal significance level (0.05) in the validation dataset.

### 2.4 Trait-fMRI association analysis

For each fMRI trait, we performed linear regression with 647 phenotypes, which were selected to reflect a variety of traits and diseases across different domains (Table S3). Specifically, there were 24 mental health traits (Category 100060), 10 cognitive traits (Category 100026), 12 physical activity traits (Category 100054), 6 electronic device use traits (Category 100053), 8 sun exposure traits (Category 100055), 3 sexual factor traits (Category 100056), 3 social support traits (Category 100061), 12 family history of diseases (Category 100034), 21 diet traits (Category 100052), 9 alcohol drinking traits (Category 100051), 6 smoking traits (Category 100058), 34 blood biochemistry biomarkers (Category 17518), 3 blood pressure traits (Category 100011), 3 spirometry traits (Category 100020), 20 early life factors (Categories 135, 100033, 100034, and 100072), 9 greenspace and coastal proximity (Category 151), 2 hand grip strength (Category 100019), 13 residential air pollution traits (Category 114), 5 residential noise pollution traits (Category 115), 2 body composition traits by impedance (Category 100009), 4 health and medical history traits (Category 100036), 3 female specific factors (Category 100069), 1 education trait (Category 100063), 48 curated disease phenotypes based on Dey et al. (2020), and 386 disease diagnoses coded according to the International Classification of Diseases (ICD-10, Category 2002). We selected all diseases in Category 2002 that had at least 100 patients in our resting fMRI imaging cohort.

For all traits, we adjusted for the effects of age (at imaging), age-squared, sex, age-sex interaction, age-squared-sex interaction, imaging site, head motion, head motion-squared, brain position, brain position-squared, volumetric scaling, height, weight, body mass index, heel bone mineral density, and the top 10 genetic principal components. Similar to the age and sex analysis, we used the UKB white British subjects in phases 1 to 3 data (*n *= 33,795 for resting and 28,907 for task) as our discovery sample and validated our results in the hold-out independent validation dataset (*n *= 5,961 for resting and 4,884 for task, removed relatives of the discovery sample). We reported *P* values from the two-sided t test and prioritized the results that were significant at the false discovery rate (FDR) level of 5% in the discovery dataset and were also significant at the nominal significance level (0.05) in the validation dataset. In comparison to the conservative Bonferroni correction, the popular FDR multiple testing procedure (Benjamini & Hochberg, 1995) was more powerful and was consistent with the exploratory nature of our fMRI-trait analysis. Thus, we mainly used FDR multiple testing control in this paper and the subset of associations that further passed the stringent Bonferroni significance level were also provided in our website.

### 2.5 Prediction models with multiple data types

We built prediction models for fluid intelligence using multi-modality neuroimaging traits, including 64,620 resting fMRI traits, 64,620 task fMRI traits, 215 DTI parameters from dMRI (Zhao et al., 2021), and 101 regional brain volumes from sMRI (Zhao et al., 2019). After removing relatives according to Bycroft et al. (2018), we randomly partitioned the white British imaging subjects into three independent datasets: training (*n* = 20,270), validation (*n* = 6,764), and testing (*n* = 6,761). The effect sizes of imaging predictors were estimated from the training data (*n* = 20,270). We removed the effects of age, age-squared, sex, age-sex interaction, age-squared-sex interaction, imaging site, head motion, head motion-squared, brain position, brain position-squared, volumetric scaling, height, weight, body mass index, heel bone mineral density, and the top 10 genetic principal components.

We also integrated other data types into our prediction model, including genetic variants and several categories of traits studied in our trait-fMRI association analysis (Table S4). For non-neuroimaging traits, the effect sizes were estimated from all UKB white British subjects except for the ones in validation and testing data (after removing relatives). We adjusted for all the covariates listed above for neuroimaging traits, except for the imaging-specific variables including imaging site, head motion, volumetric scaling, and brain position. The genetic effects were estimated by fastGWA (Jiang et al., 2019) and were aggregated using polygenic risk scores via lassosum (Mak et al., 2017). We downloaded imputed genotyping data (Category 100319) and performed the following quality controls (Zhao et al., 2019): 1) excluded subjects with more than 10% missing genotypes; 2) excluded variants with minor allele frequency less than 0.01; 3) excluded variants with missing genotype rate larger than 10%; 4) excluded variants that failed the Hardy-Weinberg test at 1 × 10^-7^ level; and 5) removed variants with imputation INFO score less than 0.8. All non-genetic predictors (including neuroimaging traits) were modeled using ridge regression via glmnet (Friedman et al., 2010) (R version 3.6.0). All model parameters were tuned in the validation dataset, and we evaluated the prediction performance on the testing data by calculating the correlation between the predicted values and the observed ones.

## 3 Results

### 3.1 Consistency and reliability of the cerebral cortex functional organizations

We examined the consistency and reliability of functional connectivity using annotations from the Glasser360 atlas in the UKB study. As in Glasser et al. (2016), we first compared the group means of two independent sets of UKB subjects: the UKB phases 1 and 2 data (imaging data released up through 2018 (Zhao et al., 2021), *n* = 17,374 for resting and 15,891 for task) and the UKB phase 3 data (data released in early 2020, *n* = 16,852 for resting and 13,232 for task, removing the relatives of subjects in early released data). Figure S3 illustrates the consistent spatial patterns of functional connectivity across the two independent groups. Similar to previous studies of other datasets (Chaarani et al., 2021; Glasser et al., 2016; Herting et al., 2018), the group mean maps in the two independent datasets of the UKB study were highly similar, with the correlation (*r*) across the 64,620 functional connectivity being 0.996 in resting fMRI and 0.994 in task fMRI. These results may suggest that the HCP-trained parcellation can provide a set of well-defined and biologically meaningful brain functional traits in the UKB datasets.

**Fig. 2.**
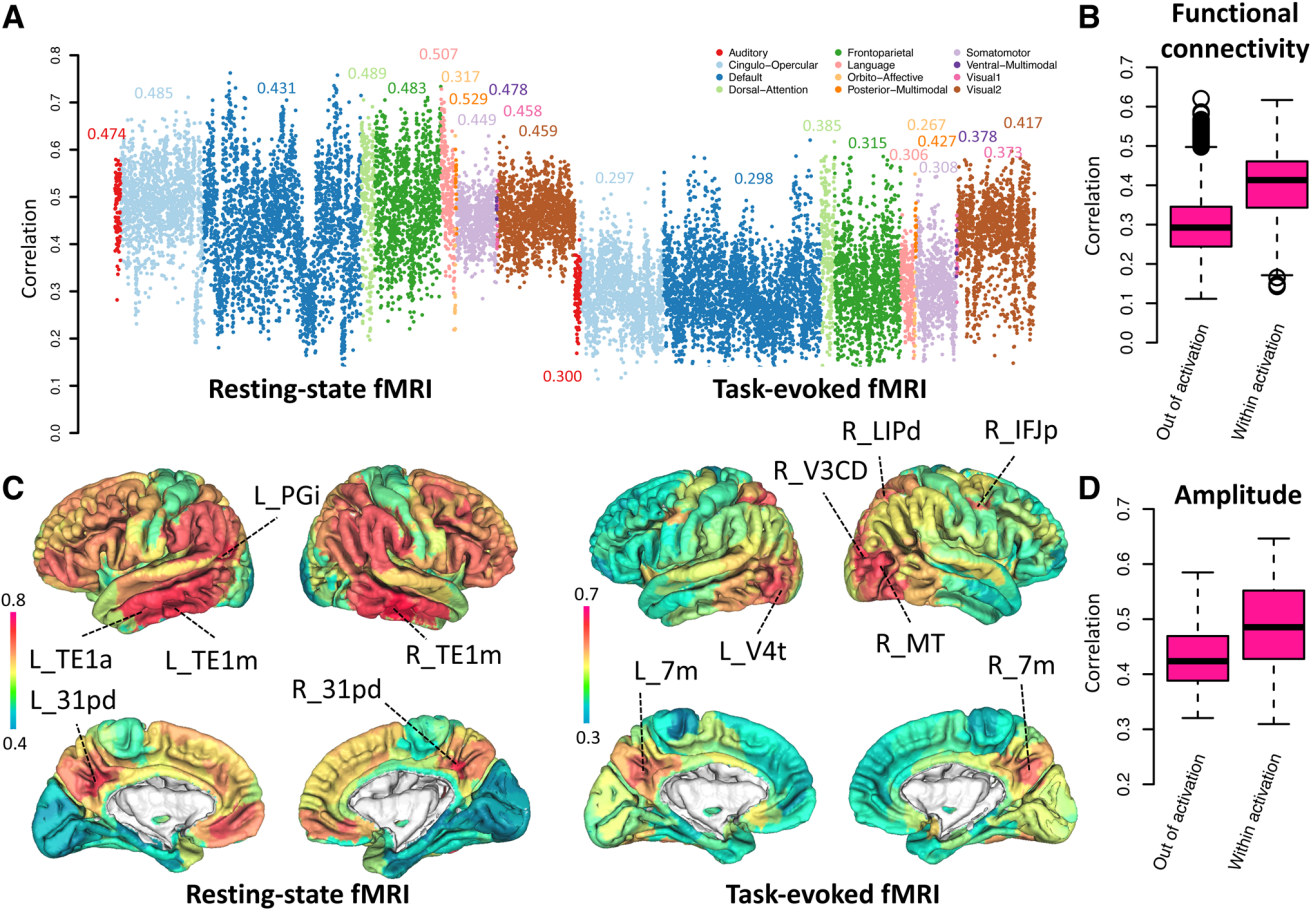
Reliability across brain functional areas and networks. (A) Comparison of reliability of functional connectivity across 12 brain functional networks in resting (left panel) and task (right panel) fMRI. (B) Comparison of reliability of functional connectivity between the activated areas (within activation) and the nonactivated areas (out of activation) in task fMRI. (C) Comparison of reliability of amplitude measures in resting (left panel) and task (right panel) fMRI. See Table S1 for information of the labeled brain areas. (D) Comparison of reliability of amplitude measures between the activated areas (within activation) and the nonactivated areas (out of activation) in task fMRI.

Next, we evaluated the intra-subject reliability of the Glasser360 atlas using the repeat scans from the UKB repeat imaging visit (*n* = 2,771 for resting and 2,014 for task, average time between visits = 2 years). We performed two analyses. The first analysis is to compare the group mean maps of the original imaging visit to those of the repeat visit. Group means were highly consistent between the two visits, with a correlation of 0.997 and 0.994 for resting and task fMRIs, respectively (ranges across different networks were [0.995, 0.999] for resting and [0.987, 0.998] for task, Fig. S4). The second analysis quantified individual-level differences between the two visits. Specifically, we evaluated the reliability of each functional connectivity by calculating the correlation between two observations from all revisited individuals. Overall, the correlation was *r *= 0.37 (standard error = 0.11) for resting fMRI and *r *= 0.31 (standard error = 0.08) for task fMRI (Fig. S5). The correlation of within-network connectivity was generally high in resting fMRI (Fig. 2A, mean *r *= 0.46). During task fMRI, the overall correlation was decreased (mean *r *= 0.32) and the secondary visual and posterior multimodal networks exhibited higher functional connectivity on average than others. In addition, the connectivity within activated functional areas (defined by group-level *Z*-statistic maps, Supplementary Note) showed a higher correlation than that within nonactivated areas (Figs. 2B and S6A, mean *r *= 0.40 vs. 0.30, *P* < 2.2 × 10^-16^). The majority of the above-defined activations occurred in the secondary visual, dorsal attention, and somatomotor networks. Furthermore, we examined the reliability of amplitude measures of fMRI (Alfaro-Almagro et al., 2018; Bijsterbosch et al., 2017; Zou et al., 2008), which quantified the functional activity within each of the 360 brain areas. The average amplitude correlation was *r *= 0.60 (standard error = 0.08) for resting fMRI and *r *= 0.45 (standard error = 0.07) for task fMRI (Fig. 2C). In accordance with the findings in functional connectivity, the reliability of amplitude measurements of activated areas in task fMRI was higher than that of nonactivated areas (Fig. 2D, mean *r *= 0.49 vs. 0.43, *P* = 1.1 × 10^-12^).

Finally, we compared the spatial patterns of UKB and HCP studies. The correlation between UKB and HCP was *r *= 0.90 for resting fMRI and *r *= 0.78 for task fMRI in the group mean analysis (Fig. S7). These results demonstrate a substantial level of overall consistency between the typical subjects in a healthy young adult cohort and those of middle age and older age. We also examined the reliability of functional connectivity in the Glasser360 atlas using the repeated scans in the HCP study (*n* = 1075, average time between two scans = 1 day). The average correlation was *r *= 0.40 (standard error = 0.09) for resting fMRI and *r *= 0.22 (standard error = 0.11) for task fMRI (the emotion task) (Fig. S6B). These results show that the two studies have similar reliability, suggesting that the quality of fMRI traits in the biobank-scale UKB study is comparable to that of the HCP project. Similar to the UKB study, the connectivity among activated functional areas (defined by group-level *Z*-statistic maps, Supplementary Note) had higher reliability than the nonactivated connectivity in HCP task fMRI (Fig. S6C, mean *r *= 0.382 vs. 0.225, *P* < 2.2 × 10^-16^). In general, the excellent group mean map consistency, as well as the similar reliability between the UKB and the HCP studies, provides confidence that the Glasser360 atlas will be able to consistently annotate the functional organization of typical subjects in a healthy population. On the other hand, the relatively low intra-subject reliability of fMRI matches previous findings (Elliott et al., 2020), which may suggest that a large sample size is needed to produce reproducible association results in downstream analyses (Marek et al., 2022).

### 3.2 Comparison of resting-state and task-evoked functional organizations

The correlation between resting fMRI and task fMRI group mean maps was 0.754 in the UKB study and 0.782 in the HCP study, indicating the high degree of similarity between intrinsic and extrinsic functional organizations (Fig. S7). Resting-task differences were observed across different networks. For example, in the auditory network, task fMRI revealed stronger intra-hemispheric connections than resting fMRI (mean = 0.482 vs. 0.314, *P* = 5.6 × 10^-11^), while the inter-hemispheric connections in task fMRI generally weakened (mean = 0.214 vs. 0.280, *P* = 8.0 × 10^-6^). Task-related changes were more complex in the default mode network. To summarize the patterns, we grouped the 77 areas in the default mode network into seven clusters, mainly based on their physical locations. We found that functional connectivity within the frontal, visual, and hippocampal clusters was stronger in task fMRI than in resting fMRI (mean = 0.314 vs. 0.384, *P* = 1.7 × 10^-9^), while the connectivity between the frontal and the other two clusters decreased (mean = 0.191 vs. 0.086, *P* < 2.2 × 10^-16^). Moreover, the frontal cluster of the default mode network can be further divided into two subclusters: the first subcluster consisted of left/right 9a, 9m, 9p, 8BL, 8Ad, and 8Av areas, mainly in the dorsolateral superior frontal gyrus (referred to as the dorsolateral superior subcluster); and the second one included left/right 10v, 10r, p32, a24, and 10d areas in the medial orbital superior frontal gyrus and pregenual anterior cingulate cortex (referred to as the medial orbital superior subcluster). The dorsolateral superior subcluster had decreased connectivity with the areas in other clusters of the default mode network in task fMRI, especially those in the temporal cluster. On the other hand, the medial orbital superior subcluster had a greater level of connectivity with a few other areas of the default mode network when performing the task, especially with the orbitofrontal complex (OFC) cluster and the neighboring 10pp area. Furthermore, the visual cluster maintained strong intra-cluster connectivity during the task, whereas its connectivity with the angular, frontal, and temporal clusters decreased (mean = 0.271 vs. 0.177, *P* < 2.2 × 10^-16^).

Several areas of the secondary visual network were less connected to other visual areas when the task was performed, including the left/right V6A (in the superior occipital), V6 (in the cuneus), VMV1 (in the lingual gyrus), and VMV2 (in the lingual and fusiform gyrus). Interestingly, some of these visual areas, such as the left/right V6, had increased functional connectivity with the default mode network. There was also an increase in connections between the default mode network and other major cognitive networks, such as the cingulo-opercular and frontoparietal. For the somatomotor network, the insula-related areas (including left/right Ig, FOP2, OP2-3, and right RI) had reduced connections with other somatomotor areas in task fMRI. Similar to the auditory network, the inter-hemispheric connectivity in the cingulo-opercular network decreased in task fMRI. Additionally, we found that the dorsal attention, frontoparietal, and language networks had similar functional connectivity patterns in resting and task fMRI. In summary, our results confirm the similarity of functional structures between resting and task fMRI, while also identifying specific patterns of differences. These network-specific patterns can be explored on our website http://fmriatlas.org/.

### 3.3 Age effects and sex differences in functional organizations

By using the large-scale fMRI data, we quantified the age and sex effect patterns on resting and task functional organizations. We used unrelated white British subjects in UKB phases 1 to 3 data release (until early 2020) as our discovery sample (*n* = 33,795 for resting and 28,907 for task) and validated the results in an independent hold-out dataset, which included non-British subjects in UKB phases 1 to 3 data release and all subjects in UKB phase 4 data release (early 2021 release, removed the relatives of our discovery sample, *n* = 5,961 for resting and 4,884 for task). The full list of the adjusted covariates can be found in the Methods section. Below, we highlighted the results passing the stringent Bonferroni significance level (7.73 × 10^-7^ = 0.05/64,620) in the discovery dataset and being significant at the nominal significance level (0.05) in the validation dataset.

There were widespread age effects on functional connectivity of resting and task fMRI, and network- and area-specific details were revealed (Fig. S8A-B). For example, as age increased, the connections within the auditory, secondary visual, somatomotor, language, and cingulo-opercular networks were generally weaker. Some areas had particularly large age effects, such as the left/right PoI2 (the posterior insular area 2) areas in the cingulo-opercular network. However, both positive and negative age effects were observed in the frontoparietal and default mode networks (Fig. S9). For example, the left/right POS2 (the parieto-occipital sulcus area 2) areas in the frontoparietal network and left/right POS1 (the parieto-occipital sulcus area 1) areas in the default mode network had strong aging effects. Negative age effects in the default mode network were strongest in the hippocampal cluster, such as the left/right PHA1 (the parahippocampal area 1) areas.

In task fMRI, age effects were different from those in resting fMRI. We highlighted a few patterns. First, the age effects in the auditory network were mainly on the inter-hemispheric connections, where the connectivity between the left and right hemispheres decreased with aging. Similarly, the inter-hemispheric connectivity between the auditory and cingulo-opercular networks declined as we aged. The age effects on intra-hemispheric connections were much weaker. Except for a few areas (such as the right 8Ad and right PEF), most areas in the cingulo-opercular and default mode networks had reduced functional connectivity with aging (Fig. S10). On the other hand, most of the functional connectivity in the secondary visual network increased with aging, especially the left/right V3A and V6A areas in the superior occipital gyrus. There were both positive and negative effects of aging on other networks, such as somatomotor, frontoparietal, and dorsal attention. Overall, these results describe the detailed age effect pattern for functional organizations at rest and during task performance.

**Fig. 3.**
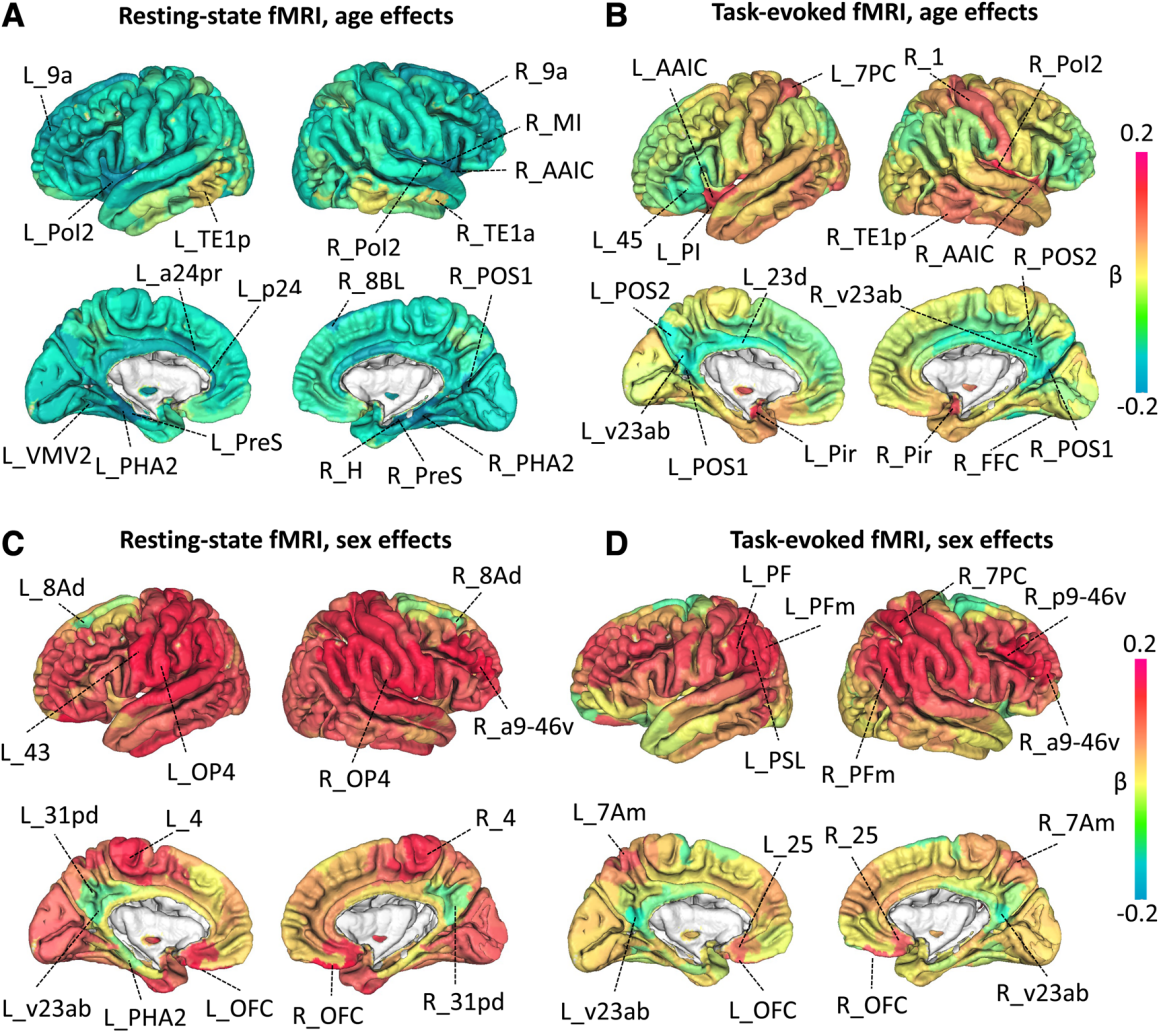
Spatial pattern of age and sex effects on brain functional organizations. We illustrate the spatial pattern of age effects on amplitude measures in (A) for resting fMRI and in (B) for task fMRI. See Table S1 for information on the labeled brain areas. (C) and (D) display the spatial pattern of sex effects on amplitude measures of resting and task fMRI, respectively. We labeled the brain areas with the strongest age and sex effects in amplitude measures.

We also examined the age effects on amplitude measures. In resting fMRI, age-related decreases in brain activity were observed in most brain areas, with the strongest effects in left and right PreS areas (the presubiculum, a subarea of the parahippocampal region, β < -0.222, *P* < 5.01 × 10^-193^, Fig. 3A). In task fMRI, however, both strong positive and negative effects on brain activity were widely observed (Fig. 3B). Because widespread age effects were detected on both functional connectivity and amplitude traits, we examined the conditional age effects on functional connectivity traits after additionally including amplitude traits as covariates. After adjusting for amplitude traits, most of the age effects on functional connectivity traits became much smaller and were not significant at the Bonferroni significance level, especially in resting fMRI (Fig. S11). For example, although a few of the strongest amplitude-adjusted age effects remained significant, most of the other moderate amplitude-adjusted age effects failed to pass the Bonferroni significance level in the default mode network. Overall, these results for amplitude traits indicate that age has a significant effect on the variation of amplitude traits across subjects, which may also be carried over to functional connectivity traits (Bijsterbosch et al., 2017).

Functional connectivity patterns differed between males and females. We found widespread sex differences across different resting fMRI networks, with the strongest differences occurring in the somatomotor network (Fig. S8C). Males had stronger functional connectivity in the somatomotor and auditory networks as well as a few specific areas, including the left/right VIP (in the superior parietal gyrus), LIPv (in the superior parietal gyrus), PH (in the inferior temporal gyrus), and V6A (in the superior occipital gyrus) of the secondary visual network, the left/right PFcm (in the superior temporal gyrus) and 43 (in the rolandic operculum) of the cingulo-opercular network, the left/right a9-46v and p9-46v (both in the middle frontal gyrus) of the frontoparietal network, and the left/right PGp (in the middle occipital gyrus) of the dorsal attention network. In the default mode network, the sex difference had a complicated pattern. Specifically, males had stronger connectivity in the hippocampal and OFC clusters, especially in the left 47m area of the posterior orbital gyrus. On the other hand, females had stronger connectivity in many other areas of the default mode network (Fig. S12).

We observed significant sex differences in task fMRI within several brain regions. These include the right V6A (located in the superior occipital gyrus) and left VMV2 (found in the lingual and fusiform gyrus) within the secondary visual network, the left/right PHA3 (situated in the fusiform gyrus) within the dorsal attention network, and the left/right RSC (located in the middle cingulate cortex) of the frontoparietal network (*P *< 7.73 × 10^-7^, refer to Fig. S13A-C). Within the language, auditory, and somatomotor networks, males exhibited stronger functional connectivity than females in numerous brain regions (see Fig. S13D-F). Additionally, males had stronger connectivity in the hippocampal and frontal areas of the default mode network, whereas females had stronger connectivity between the visual cluster and the frontal cluster (Fig. S14). As for the amplitude measures, females had stronger brain activity in many areas of the default mode network, whereas males had stronger brain activity in most other networks in resting fMRI (Fig. 3C). Sex differences were generally reduced in task fMRI amplitude measurements (Fig. 3D). Lastly, we estimated the amplitude-adjusted sex effects on functional connectivity traits by additionally controlling for the amplitude traits as covariates. Similar to the findings of the age effects, the majority of amplitude-adjusted sex effects on functional connectivity traits can be explained by amplitude traits, such as in the somatomotor and default mode networks (Fig. S15).

### 3.4 An atlas of trait associations with cerebral cortex functional areas

We aimed to explore the associations between resting and task functional organizations and 647 phenotypes. Similar to the age and sex analyses, we used unrelated white British subjects in UKB phases 1 to 3 data release as the discovery sample (*n* = 33,795 for resting and 28,907 for task) and validated the results in an independent hold-out dataset (*n* = 5,961 for resting and 4,884 for task). We prioritized significant associations that survived at the FDR 5% level in the discovery sample and remained significant at the nominal significance level (0.05) in the validation sample. Among the 647 traits, 120 had at least one significant association with resting fMRI functional connectivity measures, among which 82 further survived the Bonferroni significance level (7.73 × 10^-7^, 0.05/64,620) (Table S3). We detail below the patterns of associations relating to mental health, cognitive function, and disease status. For the complete set of results, please visit http://165.227.92.206/traitList.html.

**Fig. 4.**
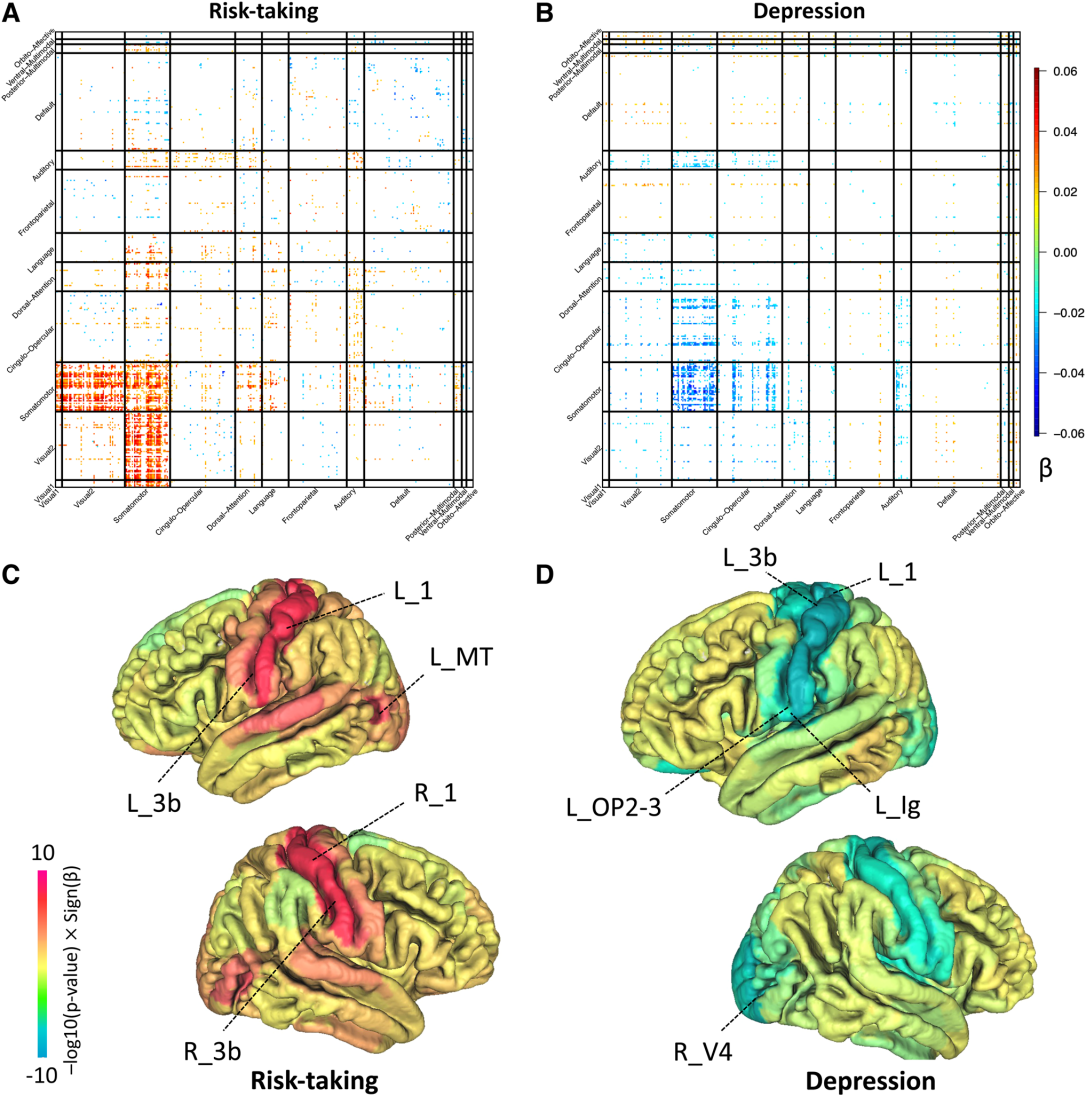
Selected complex traits that were associated with brain functional organizations. (A) Associations between risk-taking (Data field 2040) and functional connectivity of resting fMRI. This figure and the top-ranked brain areas can be viewed in an interactive version at http://165.227.92.206/trait/trait85.html. (B) Associations between depression (curated disease phenotype) and functional connectivity of resting fMRI. This figure and the top-ranked brain areas can be viewed in an interactive version at http://165.227.92.206/trait/trait230.html. We illustrated the estimated correlation coefficients that were significant at FDR 5% level in the discovery sample (*n* = 33,795) and were also significant at the nominal significance level (0.05) in the validation dataset (*n* = 5,961). (C) and (D) display the spatial pattern of associations with amplitude measures of resting fMRI for risk-taking and depression, respectively. Brain areas with the strongest associations were labeled. See Table S1 for information on these areas.

We observed strong associations between resting fMRI and multiple mental health traits, including risk-taking, depression, MDD, and neuroticism. Enrichments in specific networks and brain areas were observed. For example, risk-taking (Data field 2040) was strongly positively associated with the somatomotor network and the connections between the somatomotor and visual networks (Fig. 4A). Risk-taking was also negatively associated with the functional connections of the default mode network. Functional connectivity of sensory/motor areas was recently found to be positively associated with risk-taking (Rolls et al., 2022), and our findings were consistent with the “sensory-motor-cognitive” mode of brain functional amplitude changes related to aging (Smith et al., 2020). In addition, depression was mostly associated with reduced connectivity in the somatomotor and cingulo-opercular networks (curated disease phenotype based on ICD-10 codes, Fig. 4B). Consistent patterns were also observed in MDD (ICD-10 code F329), nervous feelings (Data field 1970), seen doctor for nervous anxiety tension or depression (Data field 2090), neuroticism score (Data field 20127), and suffer from nerves (Data field 2010).

**Fig. 5.**
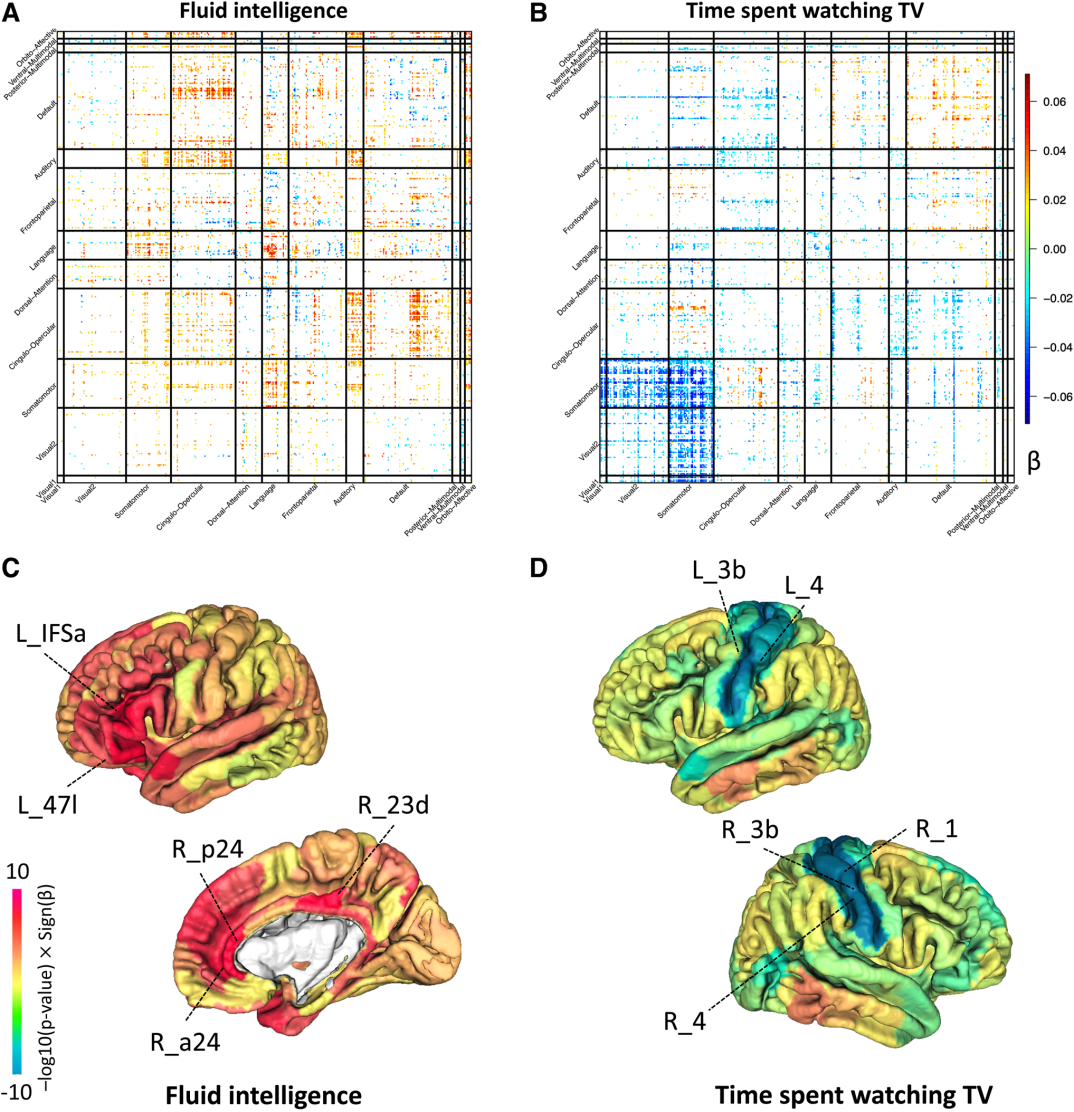
Selected complex traits that were associated with brain functional organizations. (A) Associations between fluid intelligence (Data field 20016) and functional connectivity of resting fMRI. This figure and the top-ranked brain areas can be viewed in an interactive version at http://165.227.92.206/trait/trait158.html. (B) Associations between time spent watching TV (Data field 1070) and functional connectivity of resting fMRI. This figure and the top-ranked brain areas can be viewed in an interactive version at http://165.227.92.206/trait/trait101.html. We illustrated the estimated correlation coefficients that were significant at FDR 5% level in the discovery sample (*n* = 33,795) and were also significant at the nominal significance level (0.05) in the validation dataset (*n* = 5,961). (C) and (D) display the spatial pattern of associations with amplitude measures of resting fMRI for fluid intelligence and time spent watching TV, respectively. Brain areas with the strongest associations were labeled. See Table S1 for information on these areas.

Multiple cognitive traits were associated with functional connectivity in fMRI, such as fluid intelligence (Data field 20016), the number of puzzles correctly solved (Data field 6373), duration to complete alphanumeric path (Data field 6350), and maximum digits remembered correctly (Data field 4282). These cognitive traits showed different association patterns. Fluid intelligence, for example, was associated with functional connectivity in the auditory, language, cingulo-opercular, dorsal attention, and default mode networks; most of the associations were positive (Fig. 5A). The duration to complete the alphanumeric path was mainly negatively associated with functional connectivity in the secondary visual network (Fig. S16A); the number of puzzles correctly solved was mostly related to the functional connectivity within the default mode, somatomotor, and secondary visual networks (Fig. S16B); and the maximum digits remembered correctly were positively related to the auditory and language networks (Fig. S16C). The links between brain function and several other brain-related complex traits were detected, such as the strong connections between handedness (Data field 1707) and the cingulo-opercular network (Fig. S16D). Resting functional connectivity was also widely associated with lifestyle and environmental traits, including physical activity, electronic device use, smoking, diet, alcohol, and sun exposure. For example, watching television (TV) for longer periods of time (Data field 1070) may weaken functional connectivity in the somatomotor and visual networks as well as strengthen functional connectivity in the default mode network (Fig. 5B).

Strong associations between increased functional connectivity and cardiovascular diseases were identified, including atrial fibrillation (curated disease phenotype and ICD-10 code I48), vascular/heart problems diagnosed by doctor (Data field 6150), and hypertension (curated disease phenotype and ICD-10 code I10). Atrial fibrillation is the most common clinically significant arrhythmia, and increasing evidence suggests it is associated with cognitive decline and dementia (Alonso & de Larriva, 2016). We found that atrial fibrillation was widely associated with functional connectivity across different networks (Fig. S17A-B). Hypertension and vascular/heart problems were associated with reduced functional connectivity in the auditory, somatomotor, secondary visual, and cingulo-opercular networks (Fig. S17C-D). Hypertension is a major risk factor for vascular dementia and Alzheimer’s disease and altered functional connections may reflect the early effects of vascular risk factors on brain functions (Carnevale et al., 2020).

In task fMRI, 96 traits had at least one significant association at the FDR 5% level (and significant at the nominal level in the validation dataset), and 59 further survived the Bonferroni significance level (7.73 × 10^-7^ = 0.05/64,620) (Table S3). Of the 96 traits, 69 were also significantly associated with resting fMRI at the 5% FDR level. The association patterns in task and resting fMRI were very similar for a few traits, such as atrial fibrillation (Fig. S18). For many traits, however, we observed different patterns in resting and task fMRI, including fluid intelligence (Fig. S19A-B) and the number of puzzles correctly solved (Fig. S19C-D) (*P* < 2.2 × 10^-16^). For example, both fluid intelligence and the number of solved puzzles were positively associated with intra-hemispheric connections of the auditory network in task fMRI, whereas no or negative associations were observed with inter-hemispheric connections. There were similar intra- and inter-hemispheric connection differences in the cingulo-opercular network.

We also quantified the association patterns with amplitude traits and prioritized brain areas whose functional activity was related to traits and diseases. We observed similar patterns to the functional connectivity results. For example, risk-taking has the strongest associations with the brain activity of the postcentral gyrus in the somatomotor network, especially the primary somatosensory cortex (Rolls et al., 2022) (Fig. 4C, β > 0.033, *P* < 8.14 × 10^-6^). The postcentral gyrus, insula, and Rolandic operculum areas of the somatomotor network were most negatively related to depression (Fig. 4D, β < -0.036, *P* < 7.10 × 10^-7^). All significant associations with fluid intelligence were positive, with the top three areas being the middle cingulate, anterior cingulate, and orbital part of the inferior frontal gyrus (IFG pars orbitalis) in the default mode network (Fig. 5C, β > 0.053, *P* < 1.31 × 10^-12^). Time spent watching TV was strongly negatively associated with the postcentral gyrus, precentral gyrus, paracentral lobule, and the supplementary motor area in the somatomotor network (Fig. 5D, β < -0.050, *P* < 2.03 × 10^-12^).

### 3.5 Alternative analyses using the Schaefer200 atlas

The brain parcellation may play a crucial role in the definition of the brain functional network and affect the results of downstream analysis (Popovych et al., 2021). To explore the impact of parcellation choice on the large-scale UKB study, we additionally applied another parcellation (the Schaefer200 atlas (Schaefer et al., 2018)) and repeated our analysis of the same set of subjects. Briefly, the Schaefer200 atlas partitioned the brain into 200 regions, resulting in 19,900 pairwise functional full correlation measures (200 × 199/2). We mapped the 200 regions onto the same 12 networks used in the Glasser360 atlas (Table S2).

The average reliability in the Schaefer200 atlas was *r* = 0.387 (standard error = 0.10) for resting fMRI and *r* = 0.312 (standard error = 0.07) for task fMRI, which was in the same range as the Glasser360 atlas. Figure S20 compares the reliability of the two parcellations. Glasser360 and Schaefer200 atlases showed similar patterns across a variety of networks, with the largest differences being observed in the secondary visual network, where the Glasser360 atlas was more reliable. In addition, consistent spatial patterns of functional connectivity were observed in the two parcellations, although the strength of connectivity was slightly higher in the Schaefer200 atlas, which may partly be explained by the smaller number of brain areas (Fig. S21). These results demonstrate the good generalizability of functional organizations modeled by the Glasser360 atlas.

We evaluated the age and sex effects in the Schaefer200 atlas. Figure S22 compares the age effect patterns in the Schaefer200 and Glasser360 atlases. In both atlases, decreasing resting functional connectivity was consistently associated with aging, especially in the auditory, cingulo-opercular, and somatomotor networks. The main difference was in the secondary visual network, where the age effects in the Glasser360 atlas were stronger than those in the Schaefer200 atlas. This finding may be attributed to the lower reliability of the Schaefer200 atlas in the secondary visual network, suggesting that the Glasser360 atlas may be more suitable for studying the brain connectivity of the visual cortex. In addition, consistent intra- and inter-hemispheric association differences in task fMRI were observed. The Schaefer200 and Glasser360 atlases also showed similar sex effect patterns, in which the strongest effects were both detected in the somatomotor and auditory networks (Fig. S23).

Next, we repeated the association analysis with the 647 traits. In resting fMRI, 131 traits had at least one significant association at the FDR 5% level and 83 further passed the Bonferroni significance level (2.51 × 10^-6^ = 0.05/19,900, also passing the nominal significance level (0.05) in the independent validation dataset, Table S3). Of the 120 traits with significant associations in the Glasser360 atlas analysis, 109 (90.83%) were also significant in the Schaefer200 atlas analysis. Additionally, the association maps were largely consistent in the two atlases. For example, time spent watching TV was consistently associated with decreased functional connections of the somatomotor and visual networks, as well as increased functional connectivity in the default mode network (Fig. S24A-B). Moreover, fluid intelligence was consistently linked to increased functional connectivity, particularly in the language and auditory networks (Fig. S24C-D). In both atlases, depression was associated with reduced functional connectivity in the somatomotor and cingulo-opercular networks (Fig. S25). At the FDR 5% level, 90 traits showed significant associations with task fMRI, including 76 of the 96 (79.2%) traits that were significant in the Glasser360 atlas analysis. All these results are available on our website. In summary, the Schaefer200 atlas results agree well with those of the Glasser360 atlas, indicating that the patterns observed in our Glasser360 analysis are not parcellation-specific.

Finally, we examined the trait associations with 1,701 functional connectivity traits based on the whole brain spatial ICA (Alfaro-Almagro et al., 2018; Beckmann & Smith, 2004; Hyvarinen, 1999) approach in resting fMRI. These ICA functional connectivity traits were available from the UK Biobank data release (https://www.fmrib.ox.ac.uk/ukbiobank/index.html, Data fields 25752 and 25753), which were partial correlations and the timeseries were estimated from group ICA maps via the dual regression (Alfaro-Almagro et al., 2018). Of the 647 traits, 76 demonstrated at least one significant association at the FDR 5% level and 58 remained significant at the Bonferroni significance level (2.94 × 10^-5^ = 0.05/1,701, also passing the nominal significance level in the independent validation dataset). Among the 76 ICA-significant traits, 65 (85.53%) were also significant in the above Glasser360 atlas analysis. Compared to the ICA-derived traits, parcellation-based traits from the Glasser360 atlas (which identified significant associations with 120 complex traits at the FDR 5% level and 82 at the Bonferroni significance level) were able to detect associations with more traits.

In addition, we ranked the 58 ICA-significant complex traits (at the Bonferroni significance level) by the number of their significant associations with ICA-derived traits. Then, we compared the association strengths of the top 10 traits with ICA-derived traits and those with Glasser360 traits. On these 10 traits, ICA-derived traits and Glasser360 traits showed similar levels of association strength (Fig. S26). For example, many ICA-derived and Glasser360 traits were found to be significantly associated with systolic blood pressure (Data field 4080), and most of these associations were in a similar range of effect size (Fig. S27). These results align with the results of a recent study on the functional connectome signature of blood pressure (Jiang et al., 2023). The results of Glasser360 traits indicate that the auditory and somatomotor networks may be more strongly associated with systolic blood pressure than other networks. These networks and areas may be targeted when studying hypertension-related cognitive dysfunction and brain functional damages (Carnevale et al., 2020; Naumczyk et al., 2017). In summary, parcellation-based traits may reveal more network- and area-level details with comparable association strength to ICA-derived traits.

### 3.6 Fluid intelligence prediction by integrating multiple data types

Our association analyses demonstrate the potential value of large-scale fMRI data for a variety of complex traits and disorders in clinical and epidemiological research. For example, it is of great interest to construct prediction models by integrating fMRI data and other data types (He et al., 2020; Pervaiz et al., 2020; Shen & Thompson, 2019). Fluid intelligence is a key indicator of cognitive ability and is associated with multiple neurological and neuropsychiatric disorders (Keyes et al., 2017). In this section, we performed prediction for fluid intelligence using neuroimaging traits from multiple modalities, including resting fMRI, task fMRI, diffusion MRI (dMRI) (Zhao et al., 2021), and structural MRI (sMRI) (Zhao et al., 2019). We further integrated these neuroimaging data with a wide range of other data types, including common genetic variants, biomarkers, local environments, early life factors, diet, and behavioral traits. The relative contributions and joint performance of these data types were assessed in a training, validation, and testing design. All model parameters were tuned using the validation data and we evaluated the prediction performance on the independent testing data by calculating the correlation between the predicted values and the observed intelligence, while adjusting for the covariates listed in the Methods section.

**Fig. 6:**
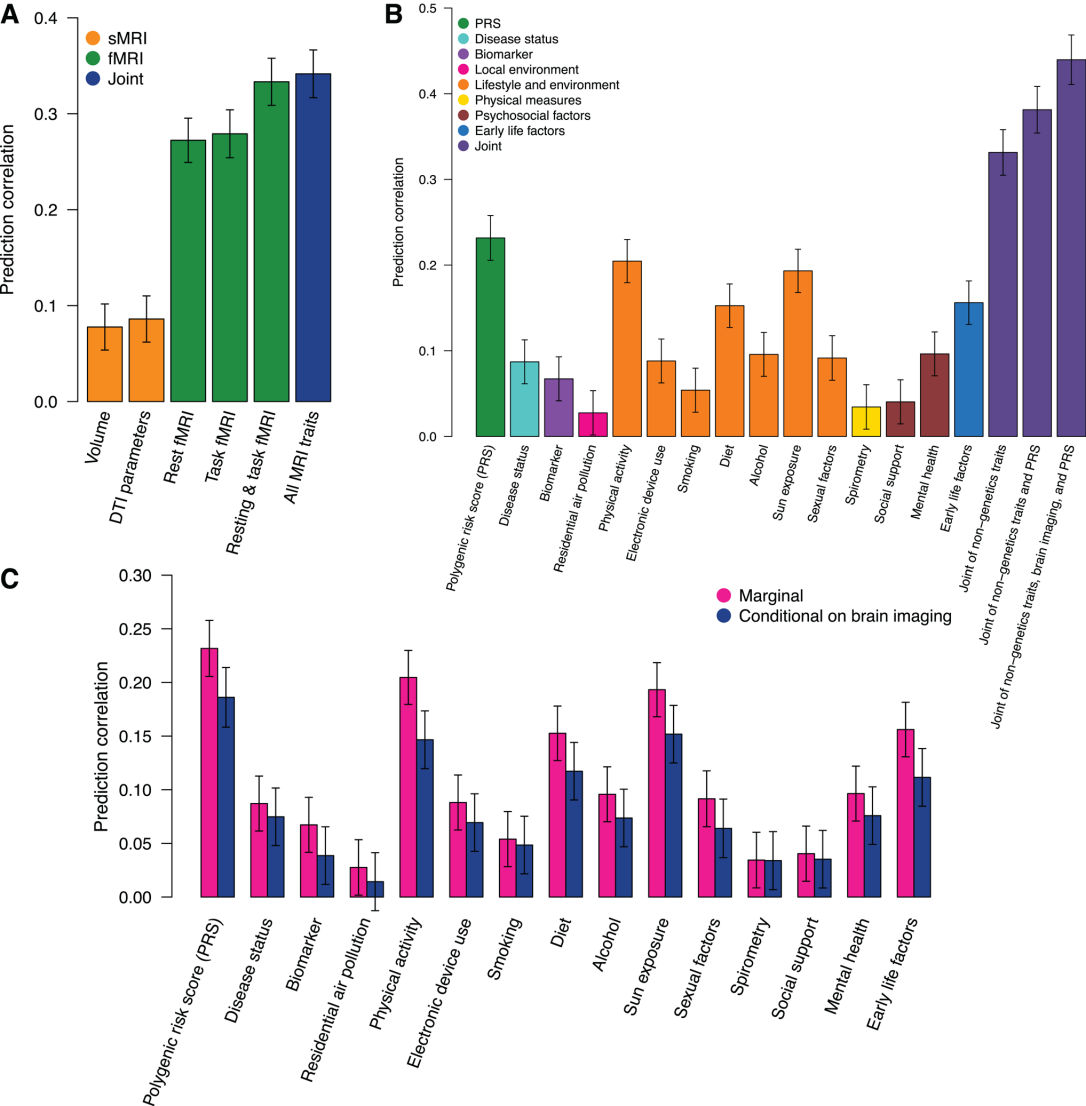
Integrative prediction model for fluid intelligence. (A) Prediction accuracy of neuroimaging traits for fluid intelligence. Volume, region brain volumes from brain structural MRI (sMRI); DTI parameters, diffusion tensor imaging parameters to measure brain white matter microstructures; All MRI traits, including brain volume, DTI parameters, resting fMRI, and task fMRI. (B) Prediction accuracy of non-neuroimaging traits from different trait categories and their joint performance. PRS, polygenic risk scores of genetic variants. (C) Comparison of predictive power of non-neuroimaging traits before (“marginal”) and after controlling for the neuroimaging traits (“conditional on brain imaging”).

The prediction performance of multi-modality neuroimaging traits was summarized in Figure 6A. The prediction correlation of resting fMRI was 0.272 (standard error = 0.012), suggesting that about 7.4% variation in fluid intelligence can be predicted by resting fMRI connectivity. The prediction correlation was similar in task fMRI (correlation = 0.279) and was improved to 0.333 by jointly using resting and task fMRI, which suggests that resting and task fMRI had different contributions to intelligence prediction. This improvement aligned with previous results reported in the HCP and Philadelphia Neurodevelopmental Cohort (PNC) studies (Gao et al., 2019), and matched our association results where both resting and task fMRI showed strong associations with fluid intelligence with different spatial patterns. In addition, the dMRI and sMRI traits had much lower prediction accuracy than fMRI traits. Specifically, the prediction correlation was 0.09 for diffusion tensor imaging (DTI) parameters of dMRI and 0.08 for regional brain volumes of sMRI. Moreover, adding these structural traits in addition to fMRI traits did not substantially improve the prediction performance (correlation = 0.342), indicating the prediction power of brain structural traits for intelligence can be largely captured by the functional traits.

Next, we examined the prediction performance of non-neuroimaging data types (Fig. 6B). The prediction correlation of intelligence genetic polygenic risk score was 0.232 (standard error = 0.013), which was slightly lower than the performance of resting fMRI. Several categories of lifestyle and environmental traits had strong predictive power, including physical activity (correlation = 0.205), sun exposure (correlation = 0.193), and diet (correlation = 0.153). Moreover, biomarkers, disease records, and early life factors all had significant predictive performance, with prediction correlations being 0.067, 0.087, and 0.156, respectively. By combining all these non-neuroimaging data types, the prediction correlation increased to 0.381. The performance was further improved to 0.440 by including neuroimaging data, which was much higher than when using only one type of data.

To explore whether the predictive power of non-neuroimaging traits can be explained by brain structural and functional variations, we evaluated their conditional predictive performance on fluid intelligence after controlling for neuroimaging traits. There was a reduction of performance on multiple categories of non-neuroimaging predictors, suggesting their effects on intelligence may be indirect and partially mediated by brain structure and function (Fig. 6C and Table S4). For example, the prediction performance of the polygenic risk score decreased from 0.232 to 0.186, indicating that 19.8% of the genetic predictive power on intelligence can be captured by brain structural and functional variations measured by brain MRI. The proportion was 28.3% for physical activity, 23.1% for diet, and 28.6% for early life factors. Overall, these results illustrate that neuroimaging traits, especially the ones from resting and task fMRI, are powerful predictors of cognitive function. Future studies can integrate genetic, biomarker, behavioral/environmental factors, and multi-modality MRI data for better prediction of brain-related complex traits and disorders.

## 4 Discussion

Inter-individual variations in brain function and their relationship to human health and behavior are of great interest. The intra-individual reliability of brain fMRI traits is generally low, although the group-level consistency is high (Chaarani et al., 2021; Elliott et al., 2020; Herting et al., 2018; Noble et al., 2021). Then, it has been suggested that a large sample size is needed for fMRI studies to detect trait associations with small effect sizes (Kennedy et al., 2021; Smith & Nichols, 2018). The UKB study provided an extensive biobank-scale data resource for quantifying fMRI associations with many phenotypes. The present study conducted a systematic analysis of intrinsic and extrinsic functional organizations with a parcellation-based approach using fMRI data collected from over 40,000 individuals. We measured differences between resting and task fMRI, investigated age and sex effects on brain function, and examined the cross-parcellation variability of our findings. We explored the fMR’s association with 647 traits chosen from a variety of trait domains. In comparison to the prior literature (Miller et al., 2016), which applied data-driven spatial ICA (Alfaro-Almagro et al., 2018; Beckmann & Smith, 2004; Hyvarinen, 1999) to about 5000 subjects, the parcellation-based approach and much larger sample size allowed us to quantify functional organizations in fine-grained details. We found distinct brain functional areas and networks that were strongly related to traits from various categories, such as mental health, physical activity, cognitive performance, and biomarkers. We developed integrative prediction models for fluid intelligence, suggesting that integrating fMRI traits with multiple data types can improve prediction performance for brain-related complex traits and diseases.

### 4.1 Resting-state and task-evoked functional organizations

The study of how the brain alters its functionality in response to tasks or stimuli is a topic of significant interest and has broad clinical applications (Zheng et al., 2022). For instance, fMRI studies involving an emotional task have consistently demonstrated abnormalities in the prefrontal cortex-limbic area among patients with anxiety disorders, who typically exhibit exaggerated responses to emotional stimuli (Li et al., 2020). Despite relatively small sample sizes, previous studies have found that intrinsic and extrinsic functional architectures share substantial similarities, with minor but consistent differences observed across various tasks (Cole et al., 2014, 2021; Gonzalez-Castillo & Bandettini, 2018; Gratton et al., 2016, 2018; Smith et al., 2009; Tavor et al., 2016). Leveraging parcellation-based data from the extensive UKB study, we corroborate that group-level intrinsic and extrinsic functional spatial patterns are largely alike (correlation = 0.754), consistent with previous fMRI datasets with smaller sample sizes (Cole et al., 2014, 2021; Gonzalez-Castillo & Bandettini, 2018; Gratton et al., 2016, 2018; Tavor et al., 2016). Moreover, we provide a more detailed analysis of resting-state functional connectivity differences. For example, our results described the complicated task-positive and task-negative functional connectivity change patterns in the default mode network. Although the default mode network has been originally recognized as brain areas with greater connectivity in resting fMRI than task fMRI (Raichle et al., 2001), recent studies have found that the default mode network also had positive functional contributions to tasks, which may result in increased activity in task fMRI (Elton & Gao, 2015).

Furthermore, our results demonstrate a remarkable spatial correlation between the UKB and HCP studies in both resting and task fMRI. This high degree of consistency across independent studies underscores the possibility of innovative joint analyses of human connectome data. Through meta-analytic amalgamation of these fMRI datasets, we have the potential to gain a more profound understanding of trait-fMRI associations' replication and enhance fMRI's predictive power for a variety of phenotypes (He et al., 2022). The integration of data from multiple sources may lead to more robust and reliable outcomes in the field of fMRI research.

### 4.2 Sex difference in fMRI

Our area- and network-specific sex effect maps can be useful for understanding sex differences in brain functional activity, as well as brain function-related cognitive impairment and brain disorders. We found that the strongest sex difference in resting fMRI was in the somatomotor network, where females had weaker functional connectivity than males (Fig. 3C). Additionally, depression was strongly associated with decreased connectivity in the somatomotor network (Fig. 4B). Considering the fact that depression is two times more prevalent in females than in males (Salk et al., 2017), our results may help understand the brain function-related sex differences in depression (Labaka et al., 2018). In addition, we found that a wide variety of complex traits were strongly associated with the functional connectivity between the visual and somatomotor networks, such as risk-taking and time spent watching TV (Figs. 4A and 5B). Future studies could investigate the biological mechanisms underlying these functional connectivity alterations as well as the causal medication pathways among lifestyle, brain function, and mental health (Zhao & Castellanos, 2016).

Additionally, our findings indicate that males demonstrated stronger task functional connectivity than females in numerous areas within the language network (Refer to Fig. S13D). This could potentially be attributable to males' more frequent use of language strategies, such as silent naming during the Hariri’s faces/shapes emotion task. On the other hand, females might rely more heavily on visual or spatial strategies. This observation calls for further investigation.

### 4.3 Trait-fMRI associations

We conducted an analysis of fMRI data alongside a range of complex traits using a discovery-validation design, generating association maps that correspond to the functional organization of the human brain during both resting and task states. These results may contribute to the development of improved disease prediction models and the identification of clinically beneficial neuroimaging biomarkers. For instance, depression and depressive mood disorders have been associated with abnormal brain connectivity across several intrinsic networks (Brakowski et al., 2017; Gudayol-Ferré et al., 2015; Korgaonkar et al., 2019). Our findings spotlight specific patterns of decreased resting functional connectivity, particularly within the somatomotor network. Extended periods of TV viewing have been linked to structural variations in the visual cortex and sensorimotor areas (Takeuchi et al., 2013). This activity has also been associated with cognitive decline (Fancourt & Steptoe, 2019) and increased dementia risk (Raichlen et al., 2022)—both closely connected with the default mode network (Grieder et al., 2018). Moreover, visual impairment and diminished functional connectivity within the visual network have been identified in Alzheimer’s disease (Huang et al., 2021; Littlejohns et al., 2022). Our results suggest that resting fMRI traits of the default mode and visual networks could serve as valuable endophenotypes for investigating the effects of environmental and lifestyle factors on aging and dementia.

The large-scale UKB data also revealed that resting and task fMRI may have different association patterns with complex traits, such as mental health and cognitive abilities. For example, depression was strongly associated with resting fMRI, but not with task fMRI. Moreover, in resting and task fMRI, the associations with fluid intelligence had different spatial distributions. Our prediction analysis further suggests that task fMRI has additional predictive power on intelligence on top of resting fMRI. These results demonstrate the differences between resting and task-evoked brain functions in terms of their connections with brain health and cognition.

### 4.4 Online resource and future development

Using the large-scale fMRI data in the UKB study, we were able to study hundreds of brain regions in a parcellation-based approach. We have utilized the rich phenotypic data in the UKB database in our fMRI-trait association analysis, which was an exploratory analysis designed to offer a publicly accessible web interface. The bioinformatics resource we have developed offers significant potential for fMRI researchers in various ways. Firstly, it allows for swift comparisons between our findings and those of existing studies within the field. Researchers can easily evaluate the congruencies or disparities in trait-fMRI associations when utilizing data from distinct studies or when identical data are analyzed by different research groups and methodologies (Botvinik-Nezer et al., 2020). Furthermore, our results can offer corroborating evidence and preliminary data for future study designs and grant proposals. Researchers can harness our findings to justify the necessity for additional data collection and the development of advanced techniques. Additionally, our resource has the potential to unearth further insights in subsequent studies through the incorporation of other fMRI data resources. For instance, conducting joint analyses with other large-scale neuroimaging studies, such as the ABCD (Chaarani et al., 2021) and CHIMGEN (Xu et al., 2020) studies, could support the replication of association findings and provide insights into age-related or cohort-related interactions throughout the lifespan. In conclusion, the online resource we have developed offers a wealth of opportunities for fMRI researchers to gain insights, compare results, support the design of future studies, and integrate with other data sources. This integration fosters an enhanced understanding and collaboration within the field.

The ongoing UKB imaging study, which aims to scan 100,000 subjects within a few years (Littlejohns et al., 2020), presents an opportunity for us to continuously update and augment our online resource. This will involve not only replicating our reported findings based on the Glasser360 and Schaefer (Schaefer et al., 2018) atlases, but also integrating additional common parcellation schemes such as the Gordon (Gordon et al., 2016), Power (Power et al., 2011), DiFuMo (Dadi et al., 2020), and data-driven ICA (Alfaro-Almagro et al., 2018; Beckmann & Smith, 2004; Hyvarinen, 1999) atlases. Moreover, we plan to explore and incorporate different data preprocessing pipelines to understand their effects on the results. For example, we will examine the effects of topographical misalignments on trait-fMRI associations and sex differences. There has been an observation in the HCP study that the cross-subject variability can be explained by the misalignment in topography between individual subjects' true connectivity topography and group-average ICA maps used by the ICA dual regression (Bijsterbosch et al., 2018, 2019). This residual functional misalignment can mean that between-subject spatial variability appears as variability in network connectivity; the extent of this problem of misinterpretation may vary across different analysis methods (e.g., group-ICA with dual-regression vs. hard parcellation). It would be interesting to quantify the effects of spatial misalignment on both parcellation-based and whole-brain ICA-based fMRI traits in the large-scale UKB dataset.

In addition, our main analyses were based on parcellation-based full correlations. Although the FMRIB's ICA-based X-noiseifier (FIX) has been applied to the UKB dataset to remove scanner artifacts and motion effects, full correlation measures can be more sensitive to the remaining global artifacts and noises than partial correlations (Feis et al., 2015; Griffanti et al., 2014). It is possible to further remove global artifacts by measuring the partial functional connectivity between paired brain regions after removing the dependency of other brain regions (Elliott et al., 2018). Future studies need to explore parcellation-based partial correlation traits for a large number of parcels (such as the 360 regions in the Glasser360 atlas) with a limited number of time points in the UKB study. Finally, we welcome user feedback and suggestions, which will help improve our project and resources to better meet the needs of the fMRI research community.

## Supplementary Material

Supplementary Material

## Data Availability

Our results and summary-level data can be downloaded and browsed at http://fmriatlas.org/. The individual-level UK Biobank data can be obtained from https://www.ukbiobank.ac.uk/. The code used in this study is available at https://zenodo.org/record/8235805.
