# Supplementary material for: An atlas of trait associations with resting-state and task-evoked human brain functional organizations in the UK Biobank
Source: Imaging Neurosci (Camb). 2023 Sep 7;1:imag-1-00015. doi: 10.1162/imag_a_00015 (PMC11105703; doi:10.1162/imag_a_00015)
Supplement: Supplementary Material [file imag_a_00015-supp.zip › P360_Supplement_Figure.pdf]

Supplementary Figures for

# An atlas of trait associations with resting-state and task-evoked human brain functional architectures in the UK Biobank

**This PDF file includes:**

Supplementary Figures Figs. S1-S27.

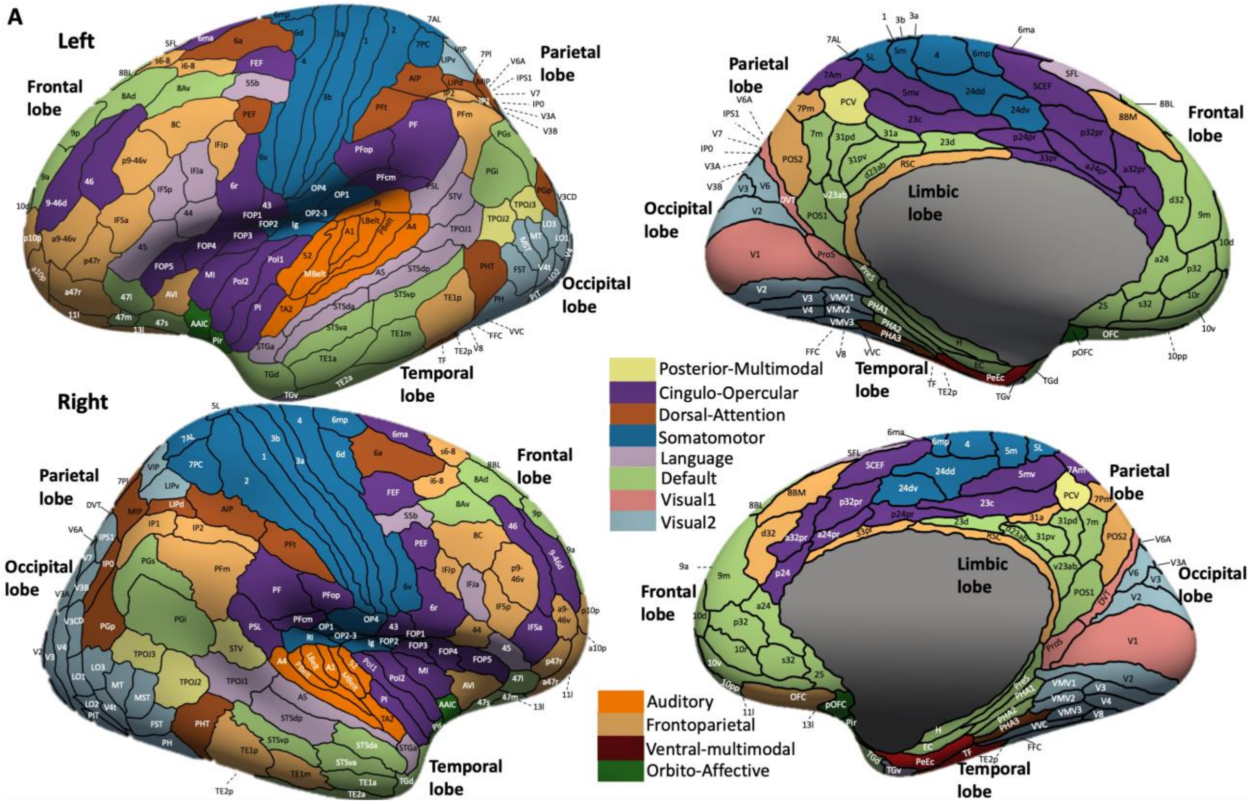


**Fig. S1 Location of the 360 functional areas defined in the Glasser360 atlas.**

See Table S1 for more information of the areas. Visual1, primary visual network; Visual2, secondary visual network; Default, default mode network.

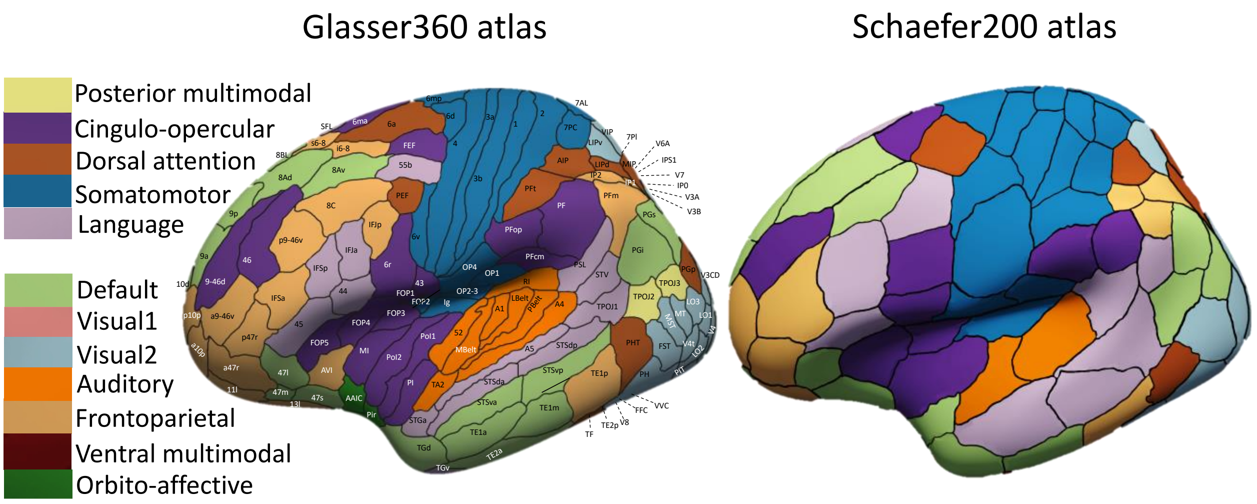


**Fig. S2 Comparison of the functional areas defined in the Glasser 360 and Schaefer200 atlases.**

See Table S2 for more information of the areas defined in the Schaefer200 atlas. Visual1, primary visual network; Visual2, secondary visual network; Default, default mode network.

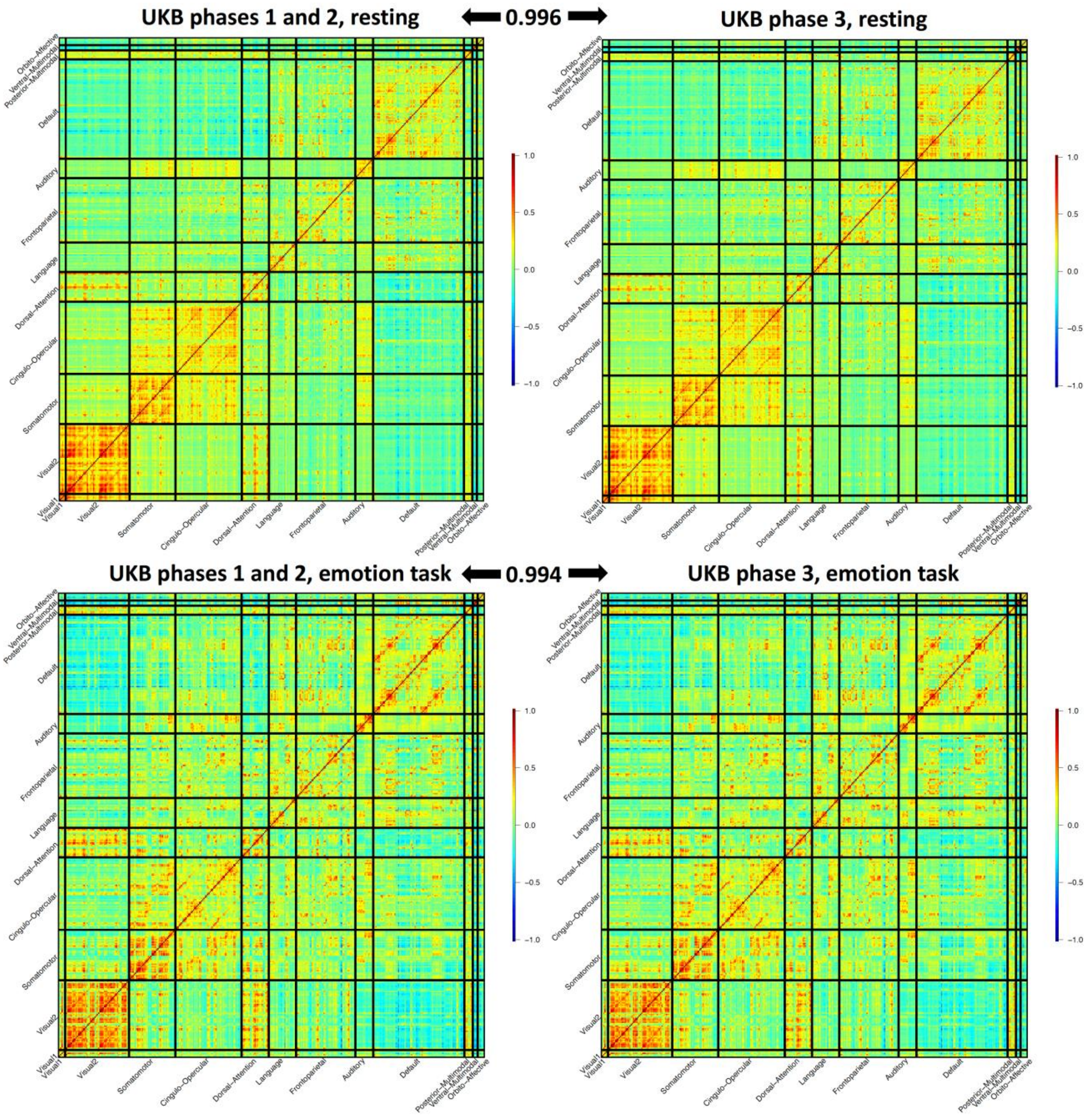


**Fig. S3 Group mean maps of fMRI in UKB phases 1 and 2 dataset (left) and UKB phase 3 dataset (right).**

The sample size in UKB phases 1 and 2 dataset was 17,374 for resting and 15,891 for task, and the sample size in UKB phase 3 dataset was 16,852 for resting and 13,232 for task (removing the relatives of subjects in early released dataset). We calculated the group average of functional connectivity for all the 64,620 (360 × 359 / 2) functional connectivity measures across all subjects within each of the two groups. The correlation of group means across all the 64620 functional connectivity measures was 0.996 for resting and 0.994 for task. Visual1, primary visual network; Visual2, secondary visual network; Default, default mode network.

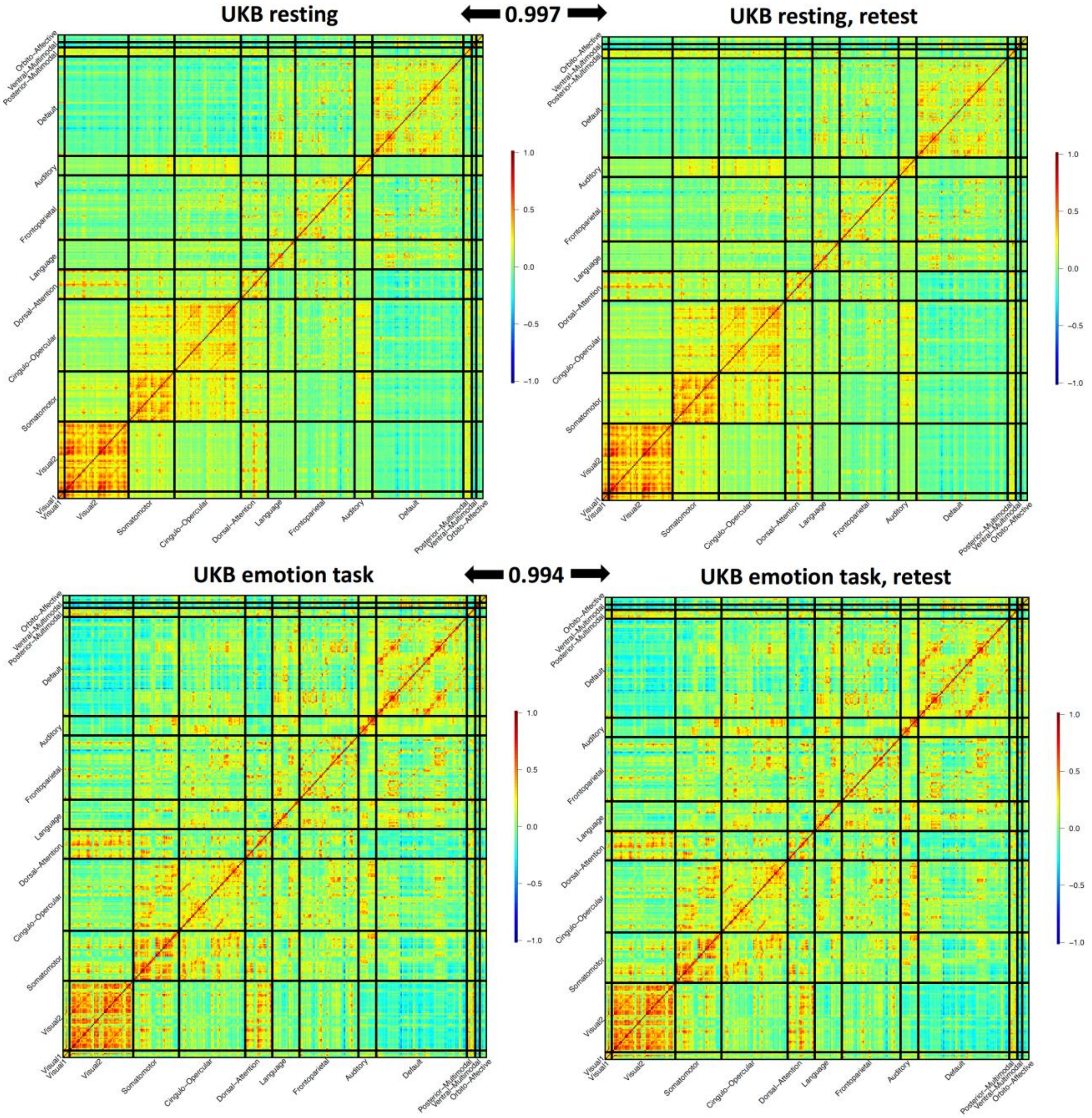


**Fig. S4 Group mean maps of fMRI in the UKB first imaging visit (left) and the UKB repeat imaging visit (right).**

The sample size of the UKB original imaging visit (UKB phases 1 to 3) dataset was 37,794 for resting and 32,144 for task, and the sample size of the UKB repeat imaging visit dataset was 2,771 for resting and 2,014 for task. We calculated the group average of functional connectivity for each functional connectivity across all subjects within each of the two datasets. The correlation of group means across all the 64620 functional connectivity measures is 0.997 for resting and 0.994 for task. Visual1, primary visual network; Visual2, secondary visual network; Default, default mode network.

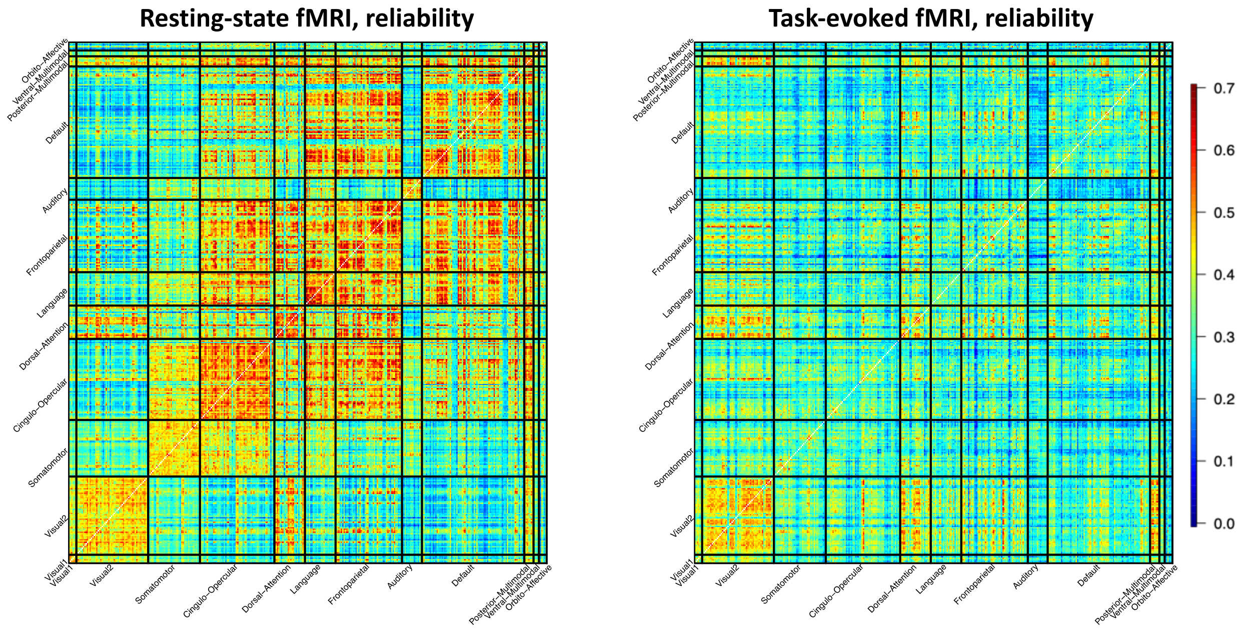


**Fig. S5 Reliability of fMRI connectivity in UK Biobank across brain functional areas and networks.**

We illustrate the spatial maps of the reliability of functional connectivity for resting fMRI (left) and task fMRI (right).

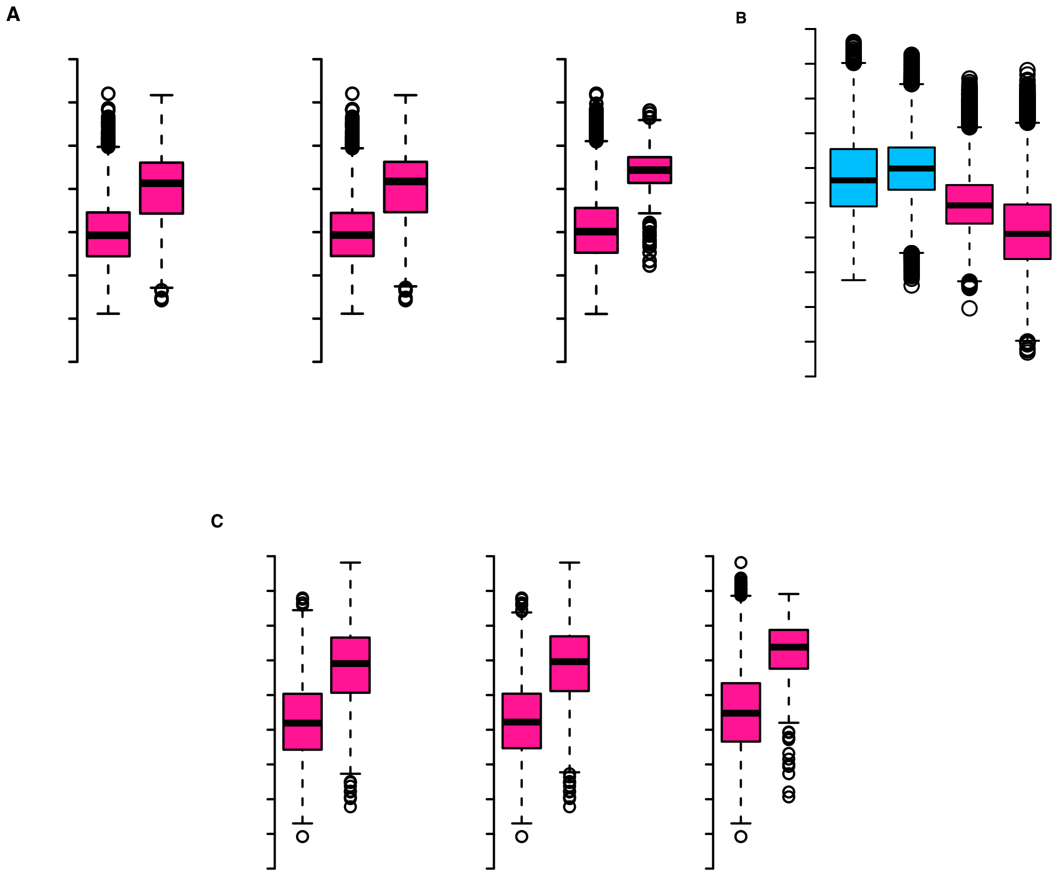


**Fig. S6 Reliability of fMRI connectivity in UK Biobank and HCP.**

**A:** Comparison of the reliability between connectivity within the activated functional areas (within activation) in UKB task-evoked fMRI and connectivity within nonactivated areas (out of activation). Shape, shape activation contrast, Face, face activation contrast, Face-shape, face-shape activation contrast. See https://biobank.ctsu.ox.ac.uk/crystal/crystal/docs/brain_mri.pdf for details. **B:** Comparison of the reliability between UKB and HCP for the resting-state fMRI (blue) and task-evoked fMRI (red). **C:** Comparison of the reliability between connectivity within the activated functional areas of HCP task-evoked fMRI and connectivity within nonactivated areas.

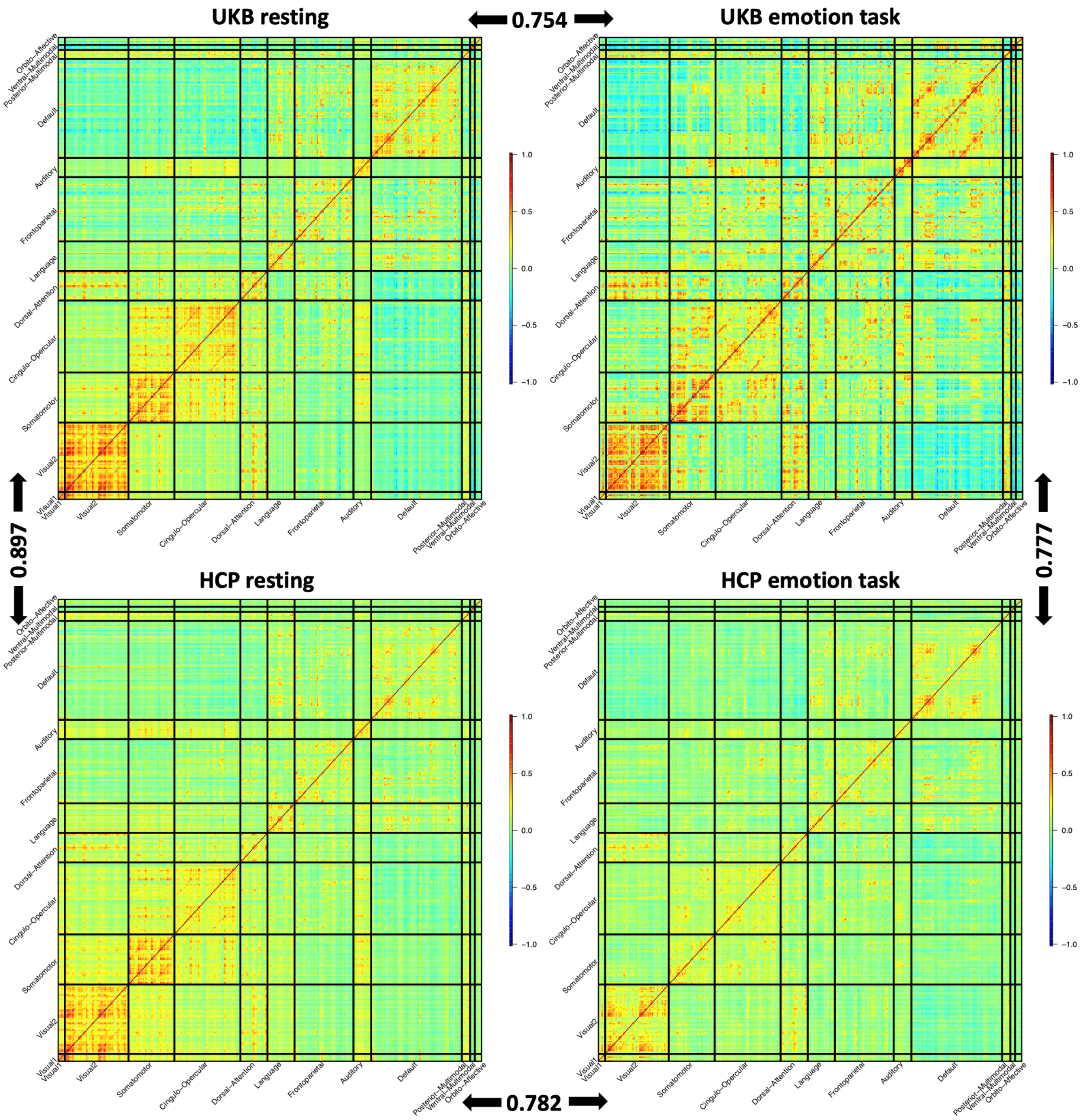


**Fig. S7 Comparison of the group mean spatial patterns in UKB and HCP studies.**

The correlation between UKB and HCP group mean maps was 0.897 for resting-state fMRI and 0.777 for task-evoked fMRI.

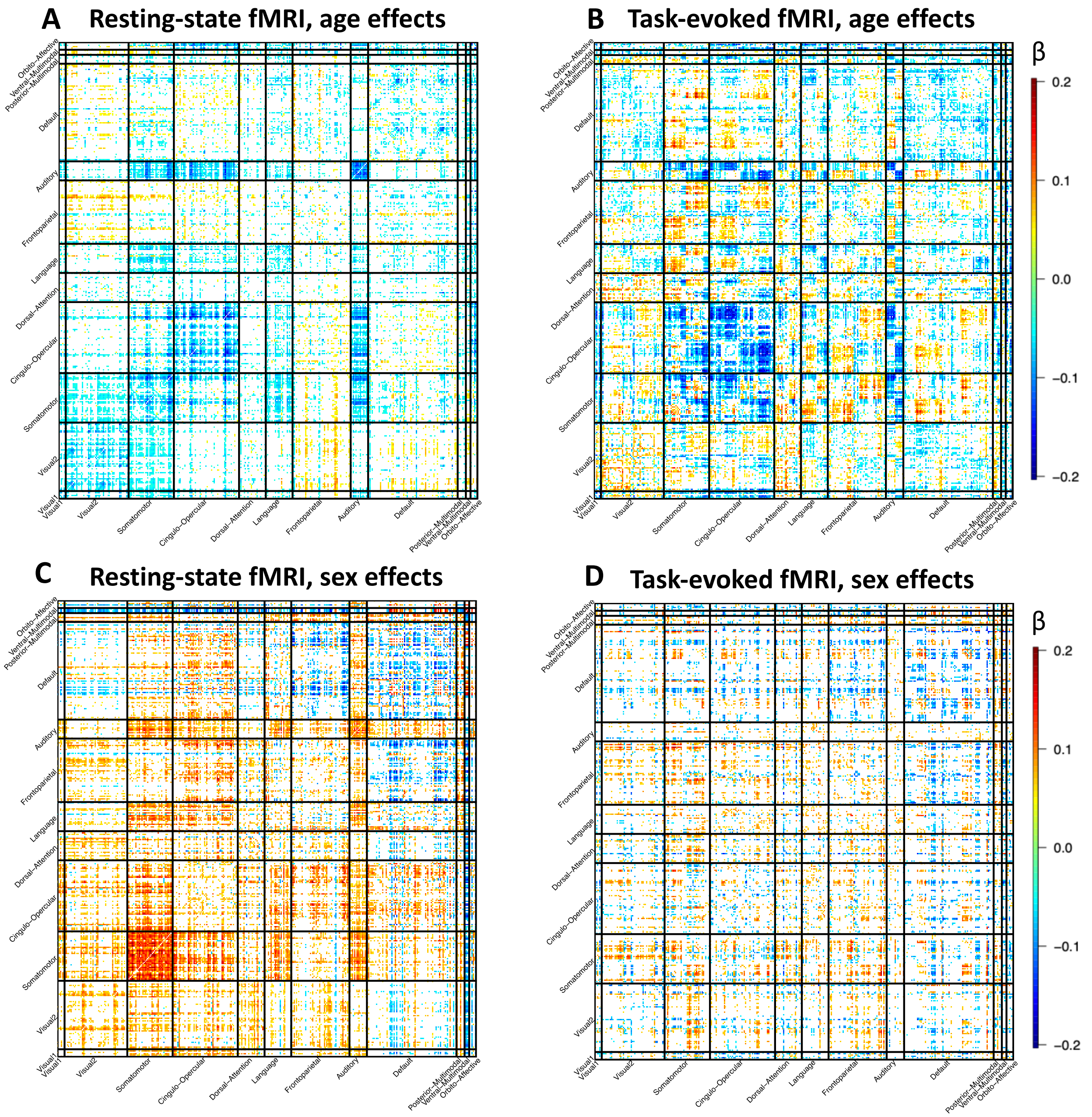


**Fig. S8 Age and sex effect patterns in resting-state and task-evoked fMRI.**

We illustrated the effects passing the Bonferroni significance level ($7.73 \times 10^{-7}$, 0.05/64,620) in the discovery dataset (*n* = 33,795) and also being significant at the nominal significance level (0.05) in the validation dataset (*n* = 5, 961).

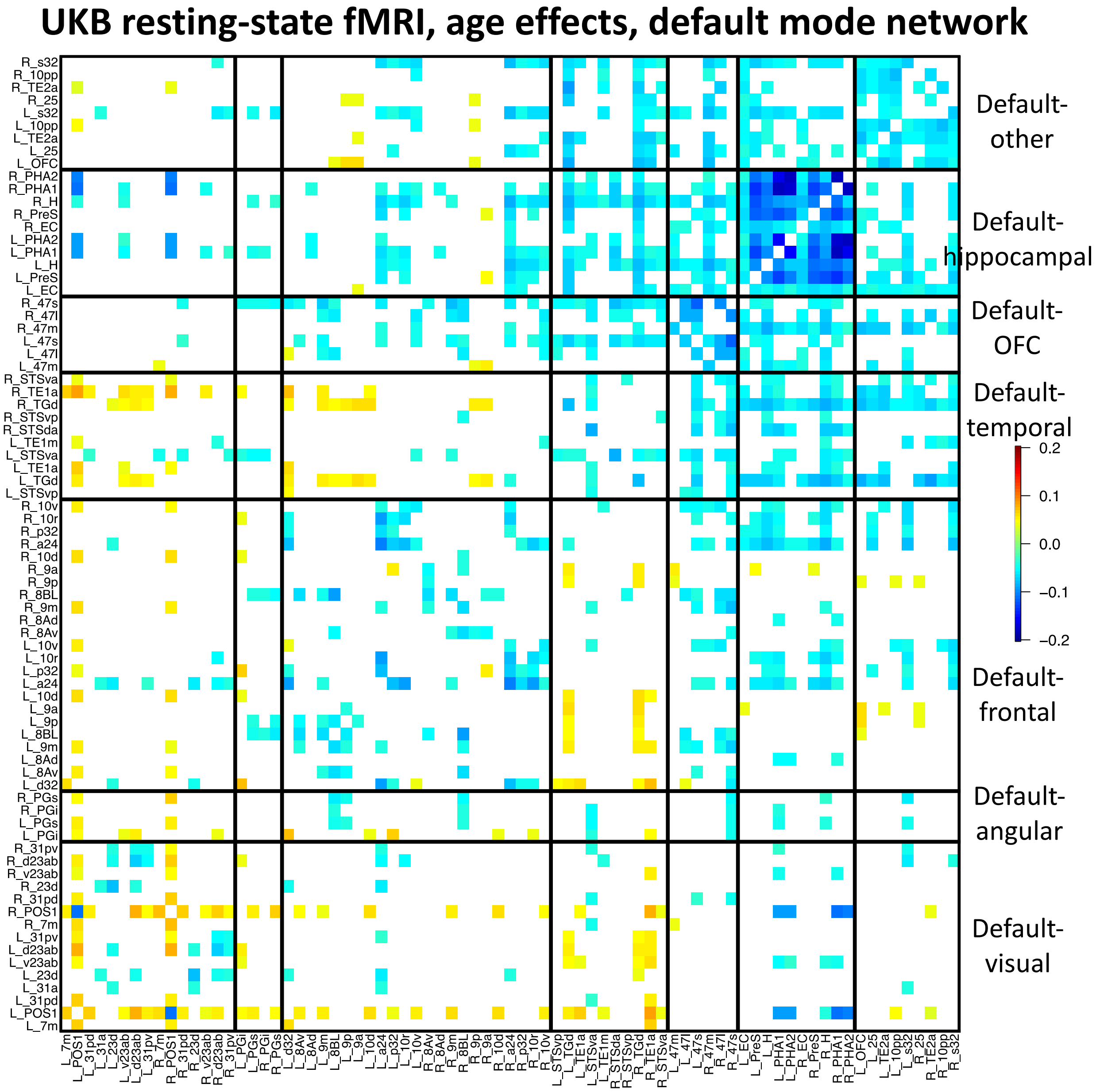


**Fig. S9 Age effect pattern in the default mode network of resting fMRI.**

We illustrated the effects passing the Bonferroni significance level ($7.73 \times 10^{-7}$, 0.05/64,620) in the discovery dataset ($n$ = 33,795) and also being significant at the nominal significance level (0.05) in the validation dataset ($n$ = 5, 961).

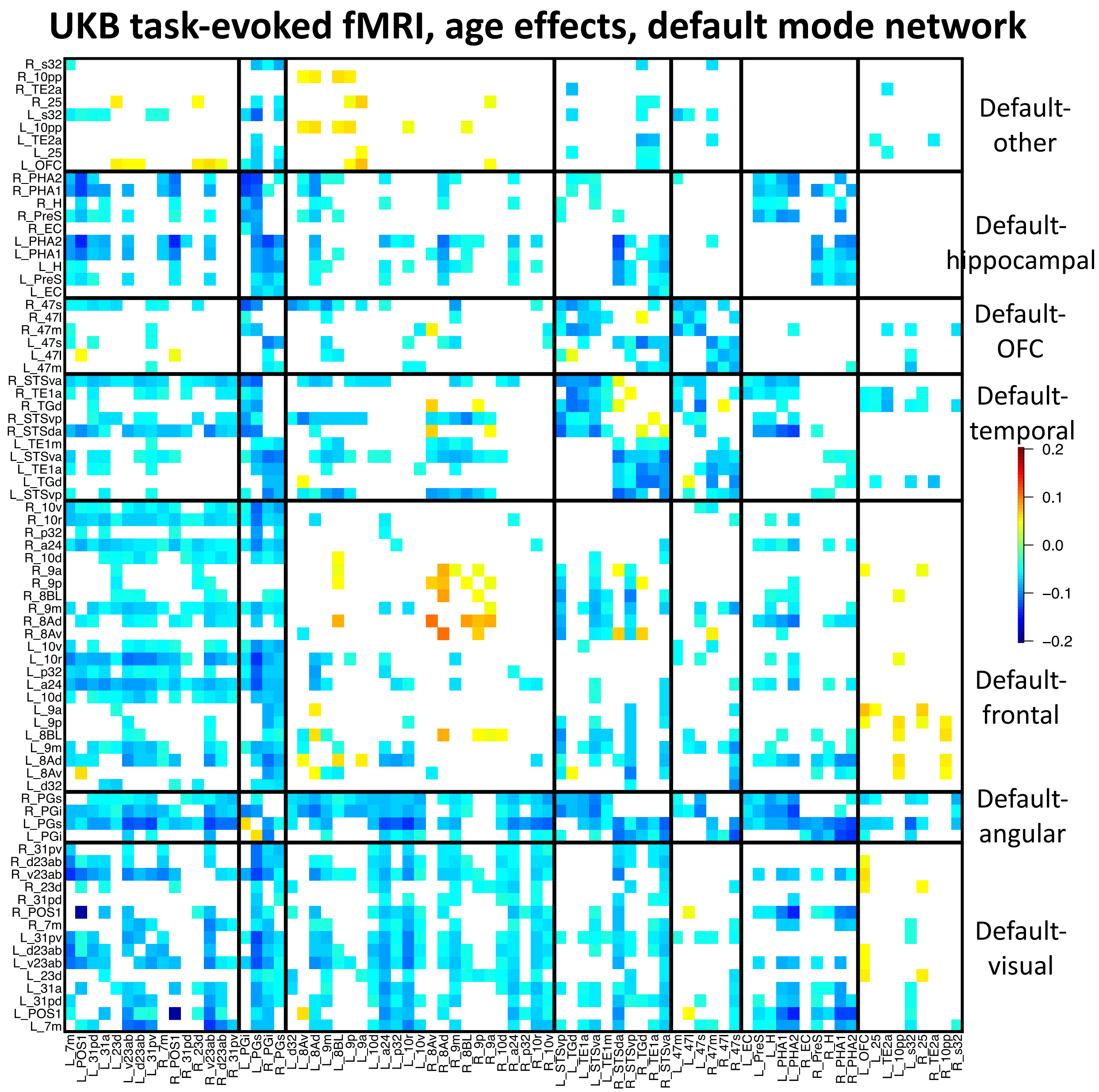


**Fig. S10 Age effect pattern in the default mode network of task fMRI.**

We illustrated the effects passing the Bonferroni significance level ($7.73 \times 10^{-7}$, 0.05/64,620) in the discovery dataset ($n$ = 28, 907) and also being significant at the nominal significance level (0.05) in the validation dataset ($n$ = 4, 884).

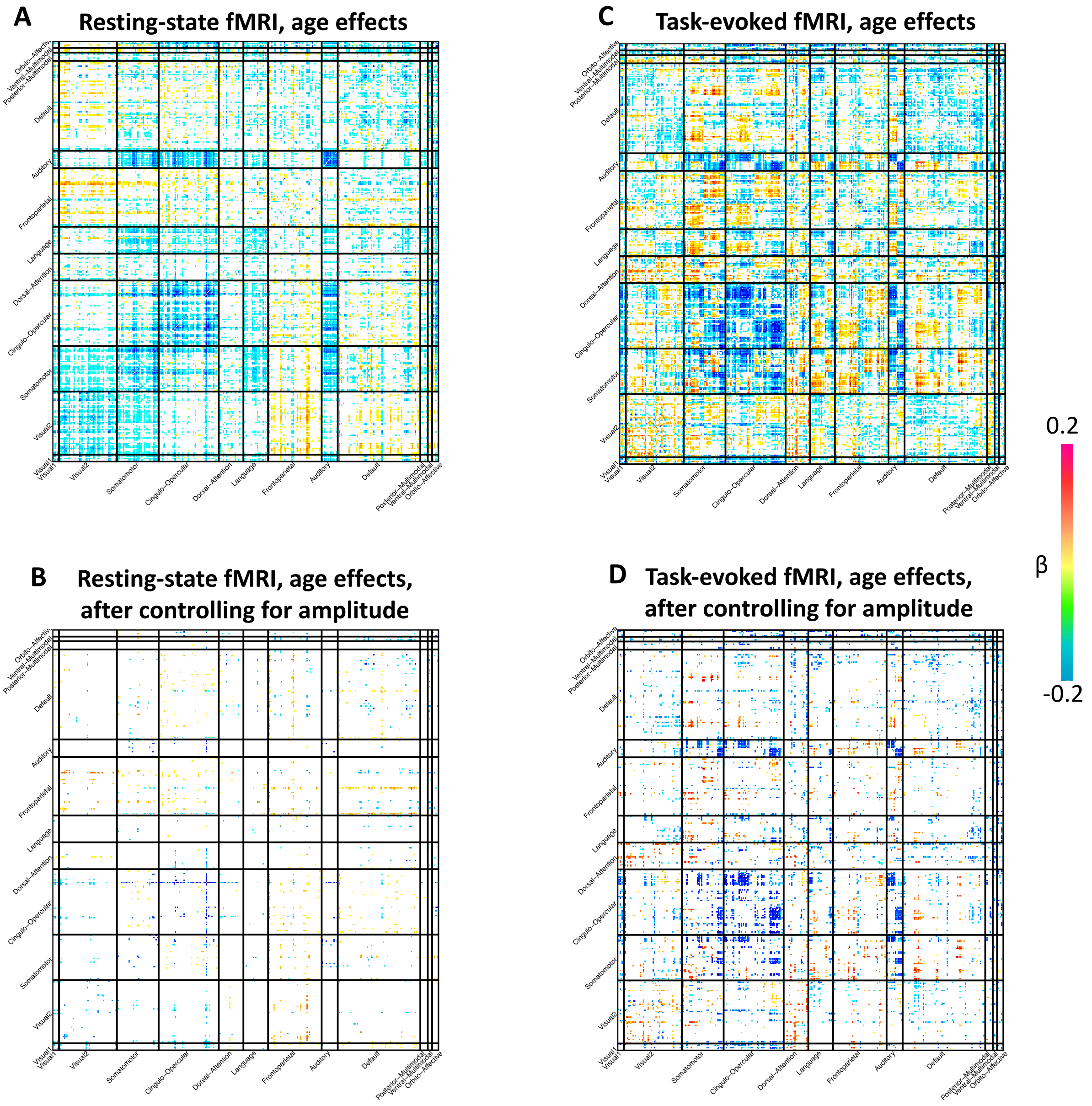


**Fig. S11 Age effect patterns before and after controlling for amplitude traits.**

In **A**, we illustrated the age effects in resting fMRI passing the Bonferroni significance level (7.73 $\times 10^{-7}$, 0.05/64,620) in the discovery dataset (*n* = 33,795) and also being significant at the nominal significance level (0.05) in the validation dataset (*n* = 5, 961). In **B**, we illustrated the remaining significant age effects in resting fMRI after additionally controlling for amplitude traits. Similarly, the panel **C** illustrated the significant age effects in task fMRI and **D** illustrated the remaining significant age effects in task fMRI after additionally controlling for amplitude traits.

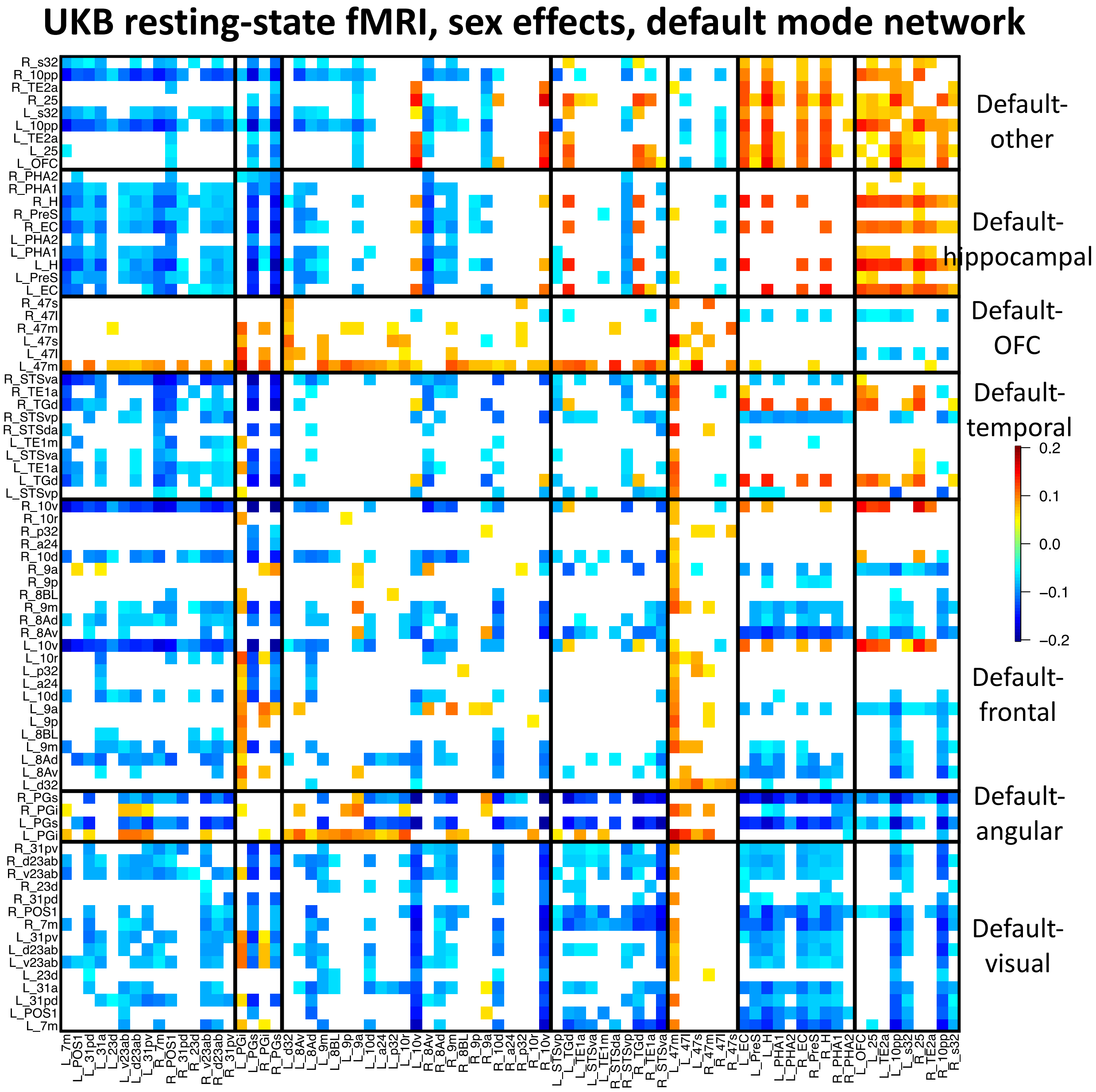


**Fig. S12 Sex effect pattern in the default mode network of resting fMRI.**

We illustrated the effects passing the Bonferroni significance level ($7.73 \times 10^{-7}$, 0.05/64,620) in the discovery dataset ($n$ = 33,795) and also being significant at the nominal significance level (0.05) in the validation dataset ($n$ = 5, 961).

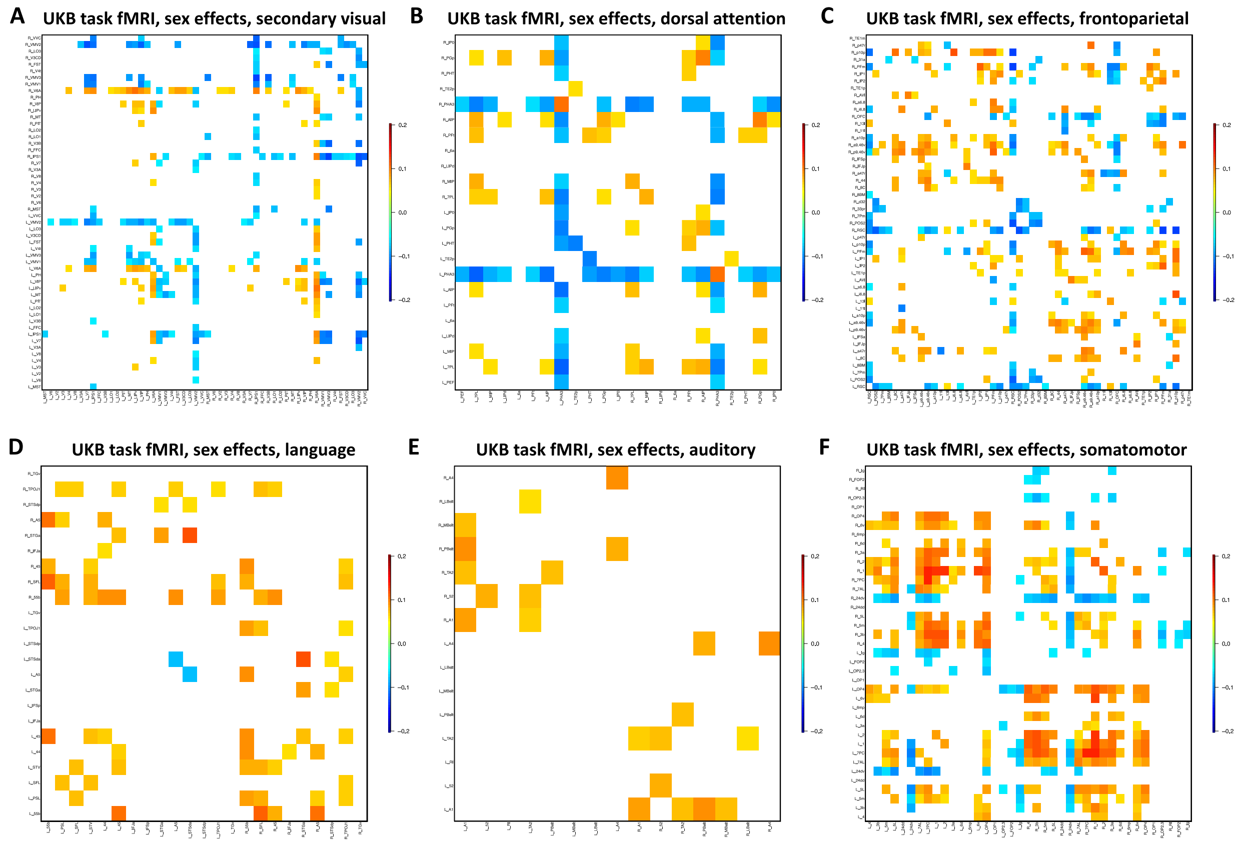


**Fig. S13 Sex effect patterns in selected functional networks of task fMRI.**

We illustrated the effects passing the Bonferroni significance level ($7.73 \times 10^{-7}$, 0.05/64,620) in the discovery dataset (*n* = 28, 907) and also being significant at the nominal significance level (0.05) in the validation dataset (*n* = 4, 884).

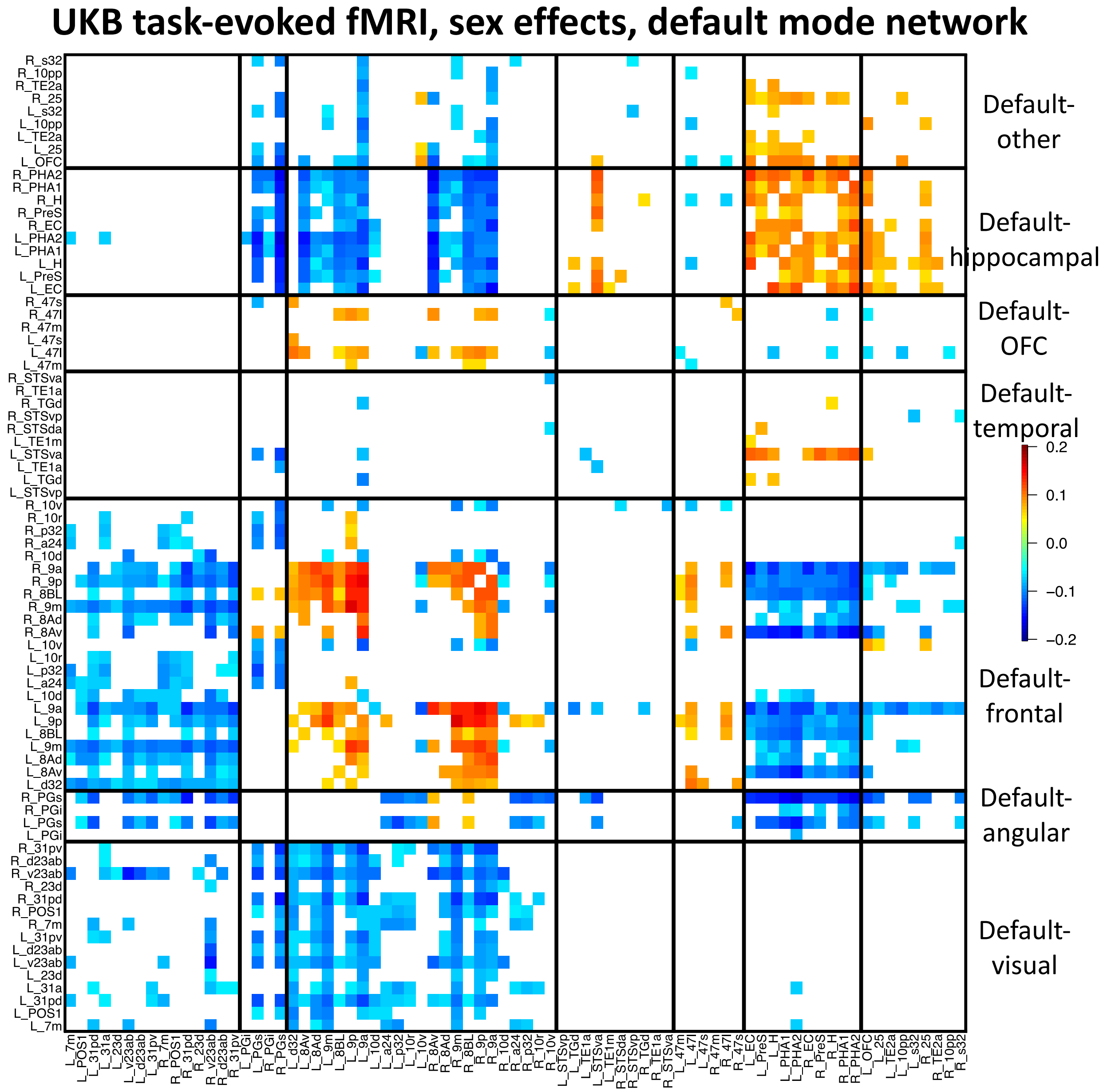


**Fig. S14 Sex effect pattern in the default mode network of task fMRI.**

We illustrated the effects passing the Bonferroni significance level ($7.73 \times 10^{-7}$, 0.05/64,620) in the discovery dataset ($n$ = 28, 907) and also being significant at the nominal significance level (0.05) in the validation dataset ($n$ = 4, 884).

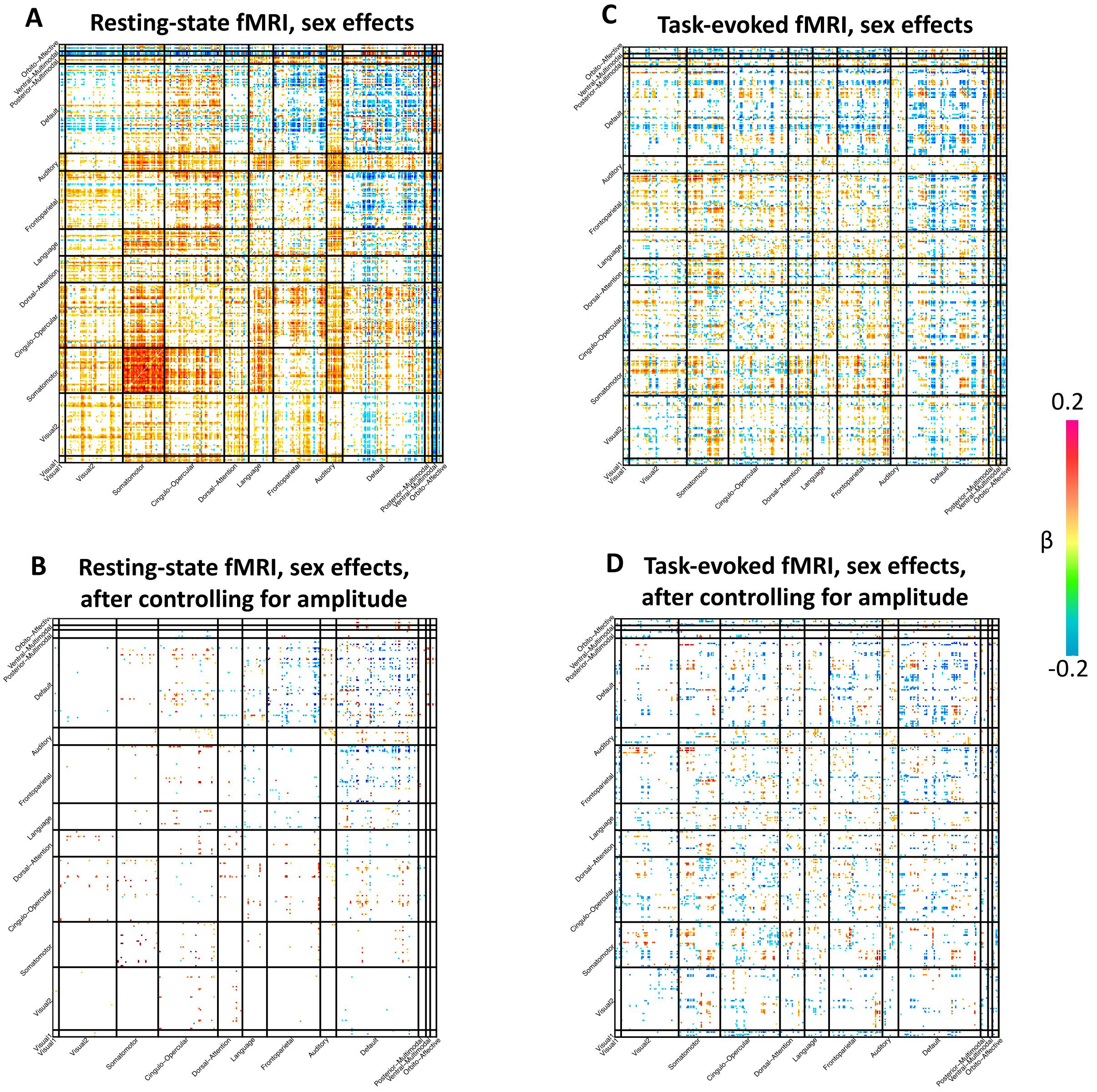


**Fig. S15 Sex effect patterns before and after controlling for amplitude traits.**

In **A**, we illustrated the sex effects in resting fMRI passing the Bonferroni significance level (7.73 × $10^{-7}$, 0.05/64,620) in the discovery dataset (*n* = 33,795) and also being significant at the nominal significance level (0.05) in the validation dataset (*n* = 5, 961). In **B**, we illustrated the remaining significant sex effects in resting fMRI after additionally controlling for amplitude traits. Similarly, the panel **C** illustrated the significant sex effects in task fMRI and **D** illustrated the remaining significant sex effects in task fMRI after additionally controlling for amplitude traits.

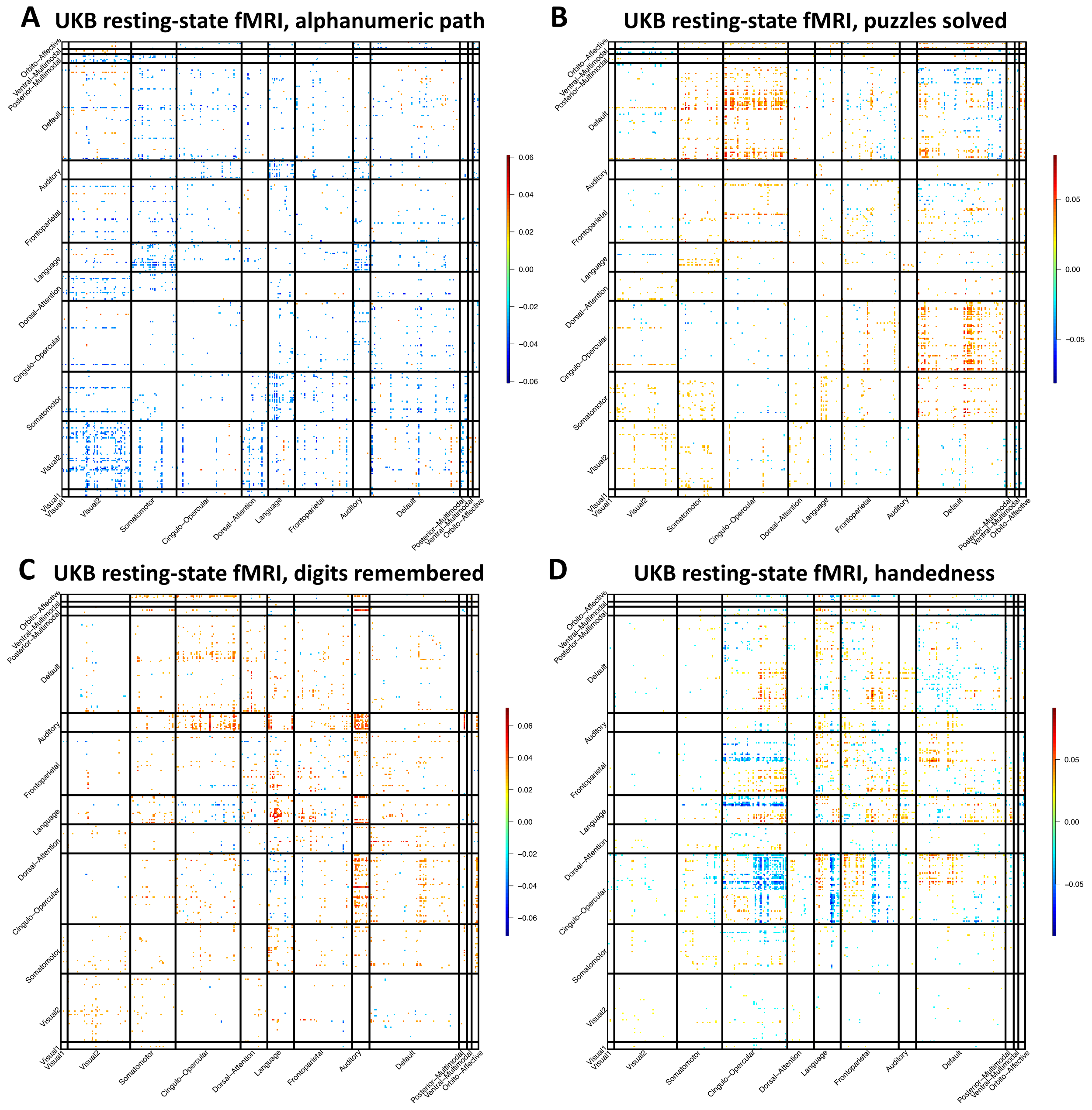


**Fig. S16 Associations between resting fMRI and selected brain-related complex traits.**

We illustrated the correlation coefficients that were significant at FDR 5% level in the discovery dataset (*n* = 33,795) and were also significant at the nominal significance level (0.05) in the validation dataset (*n* = 5, 961). Alphanumeric path, duration to complete alphanumeric path (Data field 6350); Puzzles solved, the number of puzzles correctly solved (Data field 6373); digits remembered, maximum digits remembered correctly (Data field 4282); and handedness, right or left-handed (chirality/laterality, 1=right-handed and 2=left-handed, Data field 1707)

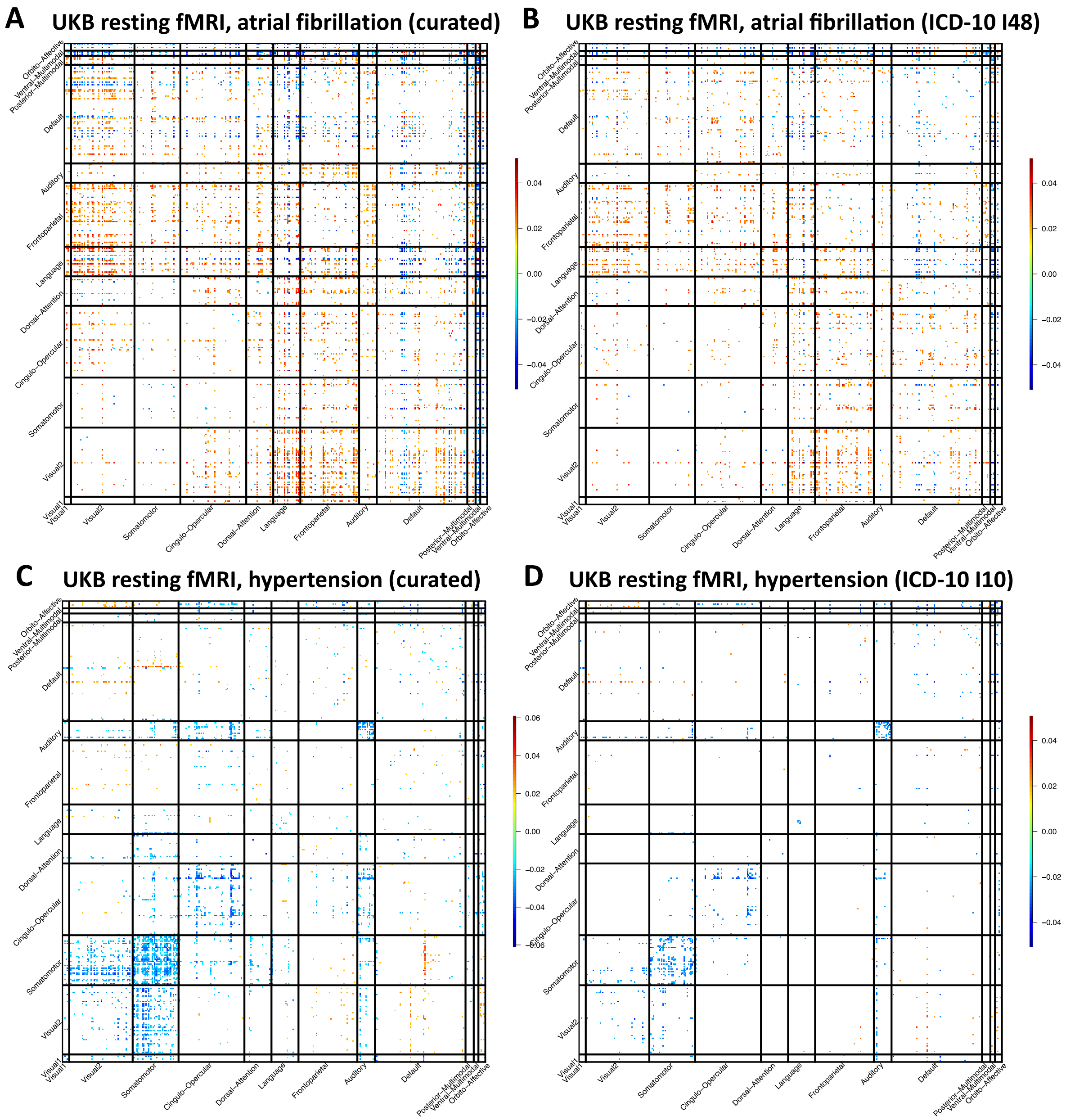


**Fig. S17 Associations between resting fMRI and selected diseases.**

We illustrated the correlation coefficients that were significant at FDR 5% level in the discovery dataset ($n$ = 33,795) and were also significant at the nominal significance level (0.05) in the validation dataset ($n$ = 5, 961). "Curated" indicates curated disease phenotype by combining multiple related ICD-10 codes.

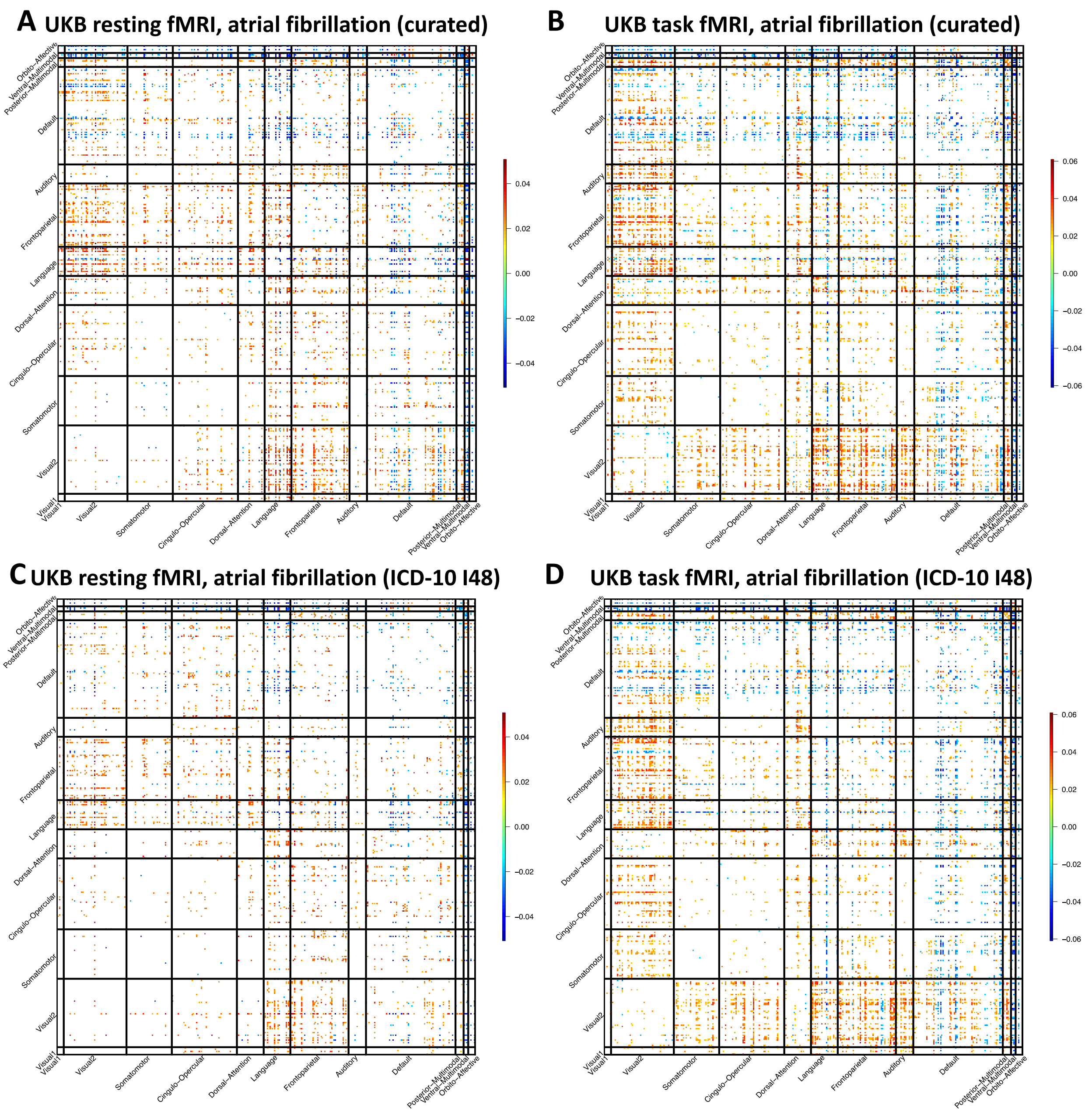


**Fig. S18 Associations between atrial fibrillation and resting and task fMRI.**

We illustrated the correlation coefficients that were significant at FDR 5% level in the discovery dataset ($n$ = 33,795 for resting and 28, 907 for task) and were also significant at the nominal significance level (0.05) in the validation dataset ($n$ = 5, 961 for resting and 4, 884 for task). "Curated" indicates curated disease phenotype by combining multiple related ICD-10 codes.

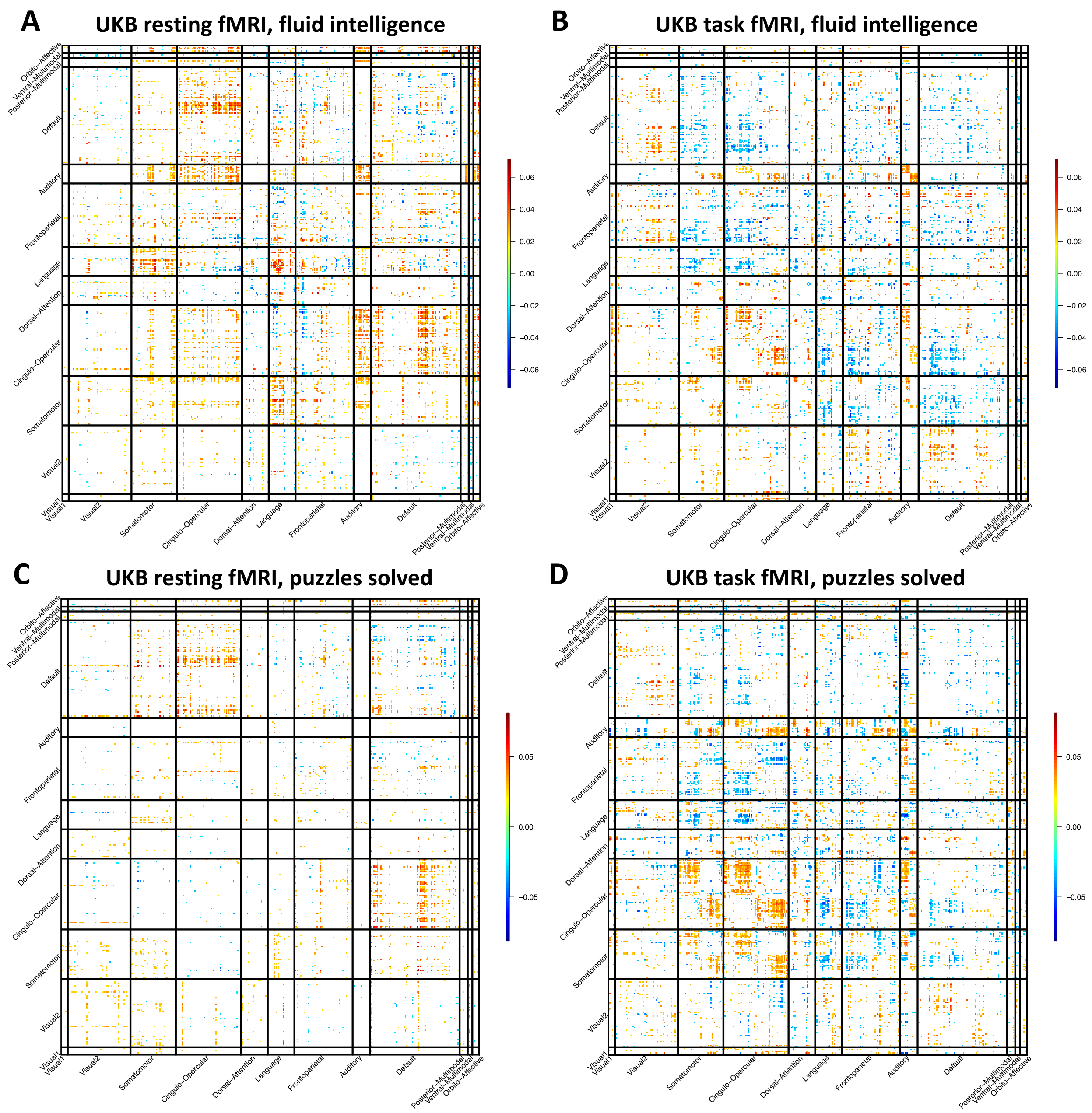


**Fig. S19 Associations between selected cognitive traits and resting and task fMRI.**

We illustrated the correlation coefficients that were significant at FDR 5% level in the discovery dataset (*n* = 33,795 for resting and 28, 907 for task) and were also significant at the nominal significance level (0.05) in the validation dataset (*n* = 5, 961 for resting and 4, 884 for task). Fluid intelligence, fluid intelligence score (Data field 20127); and puzzles solved, the number of puzzles correctly solved (Data field 6373).

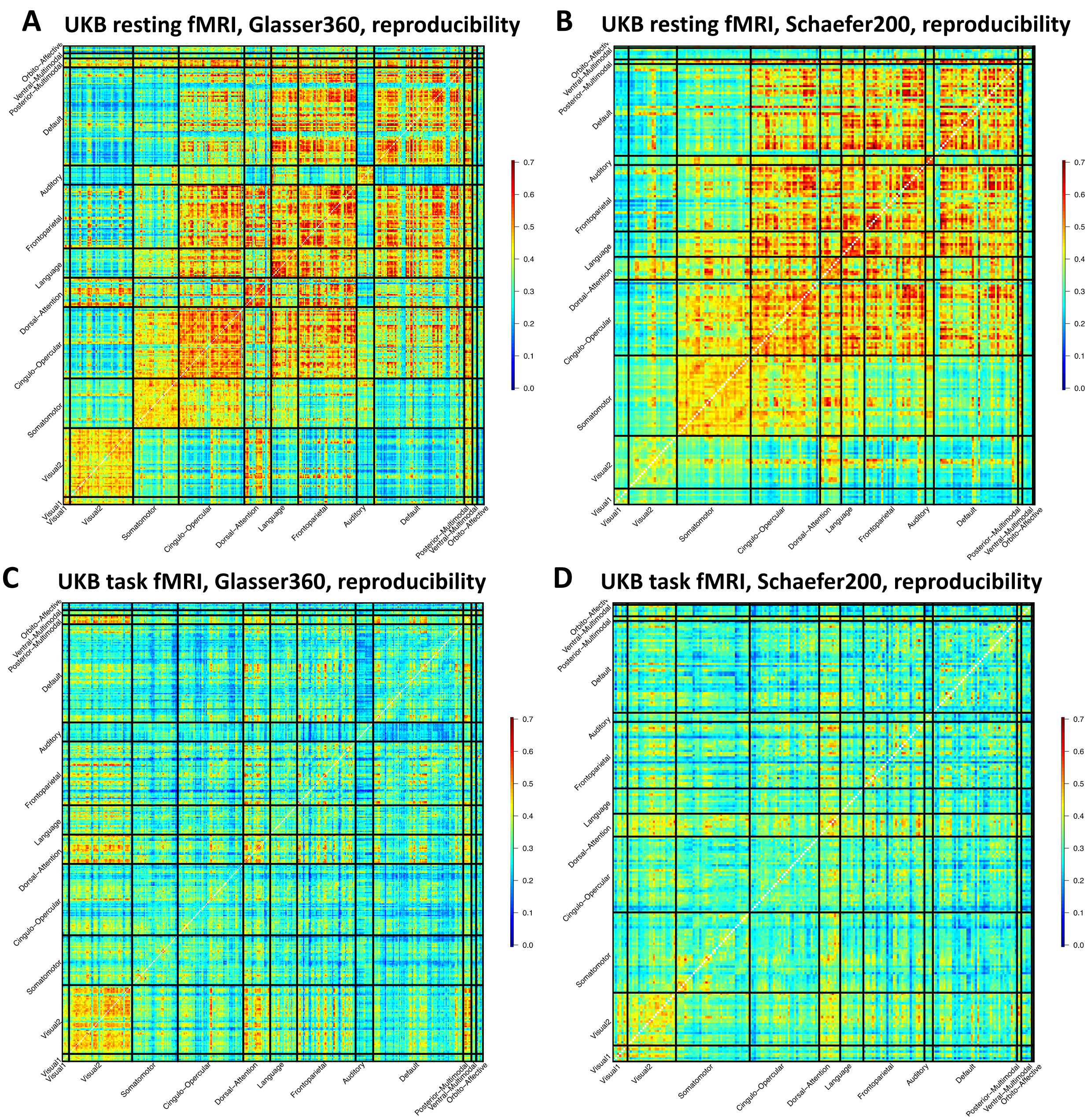


**Fig. S20 Comparison of the reliability spatial patterns in the Glasser360 and Schaefer200 atlases.**

The sample size of the UKB repeat imaging visit dataset was 2,771 for resting fMRI and 2,014 for task fMRI.

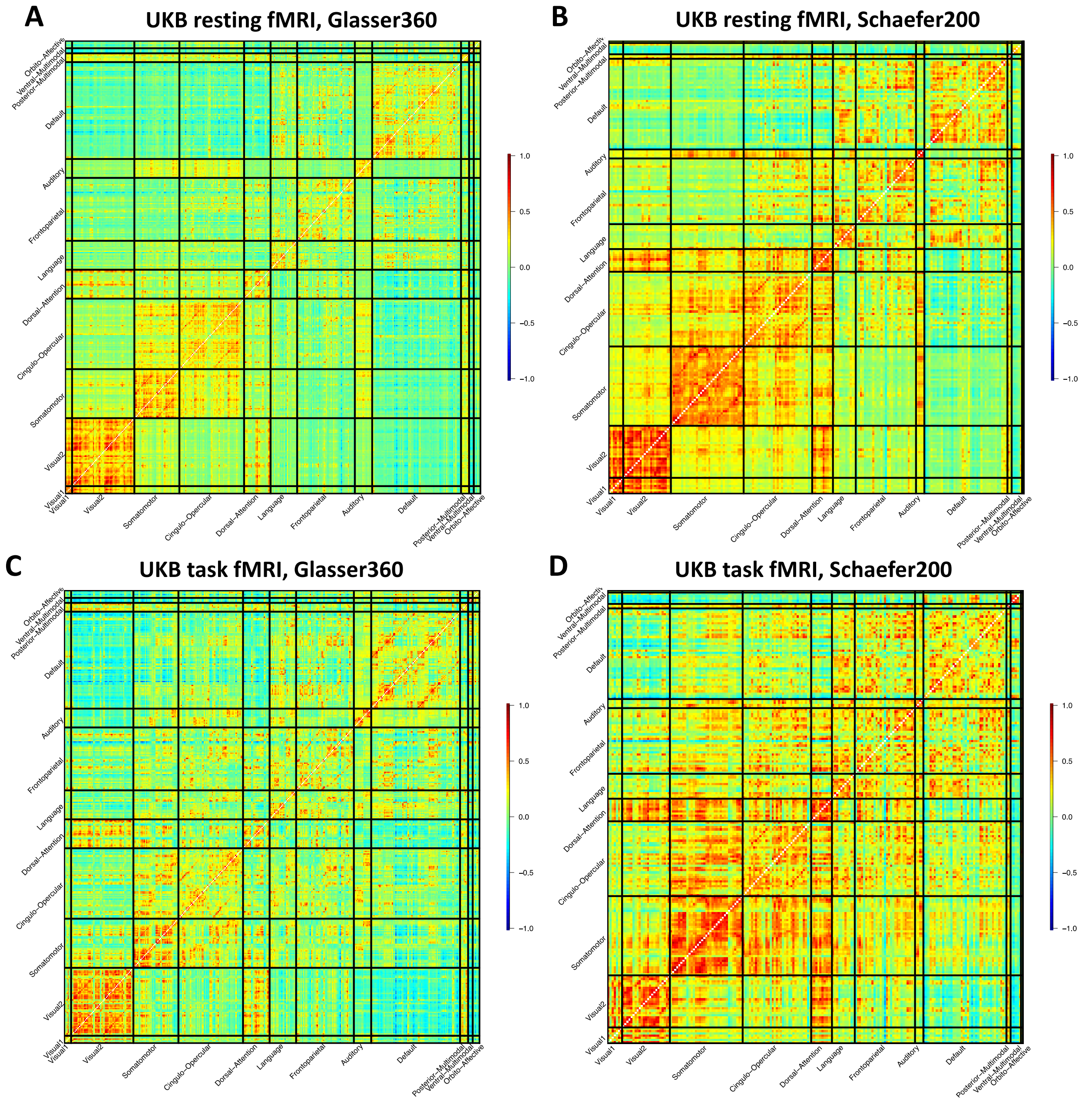


**Fig. S21 Comparison of the group mean spatial patterns in the Glasser360 and Schaefer200 atlases.**

The sample size was 37,794 subjects for resting fMRI and 32,144 subjects for task fMRI. We calculated the group average for each functional connectivity across all subjects.

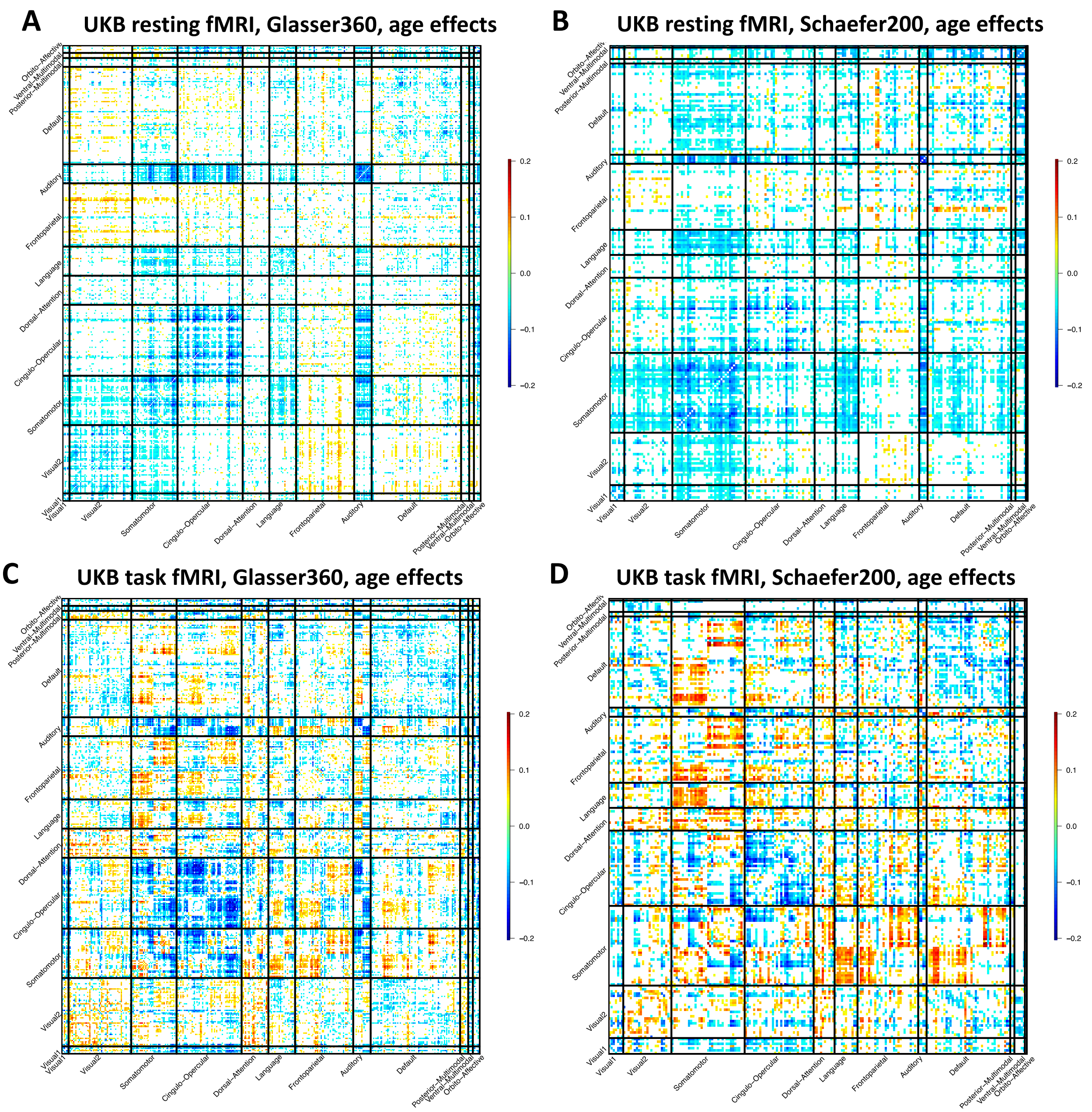


**Fig. S22 Comparison of the age effect patterns in the Glasser360 and Schaefer200 atlases.**

We illustrated the effects passing the Bonferroni significance level (64,620 tests for Glasser360 and 19,900 tests for Schaefer200) in the discovery dataset (*n* = 33,795 for resting and 28, 907 for task) and also being significant at the nominal significance level (0.05) in the validation dataset (*n* = 5, 961 for resting and 4, 884 for task).

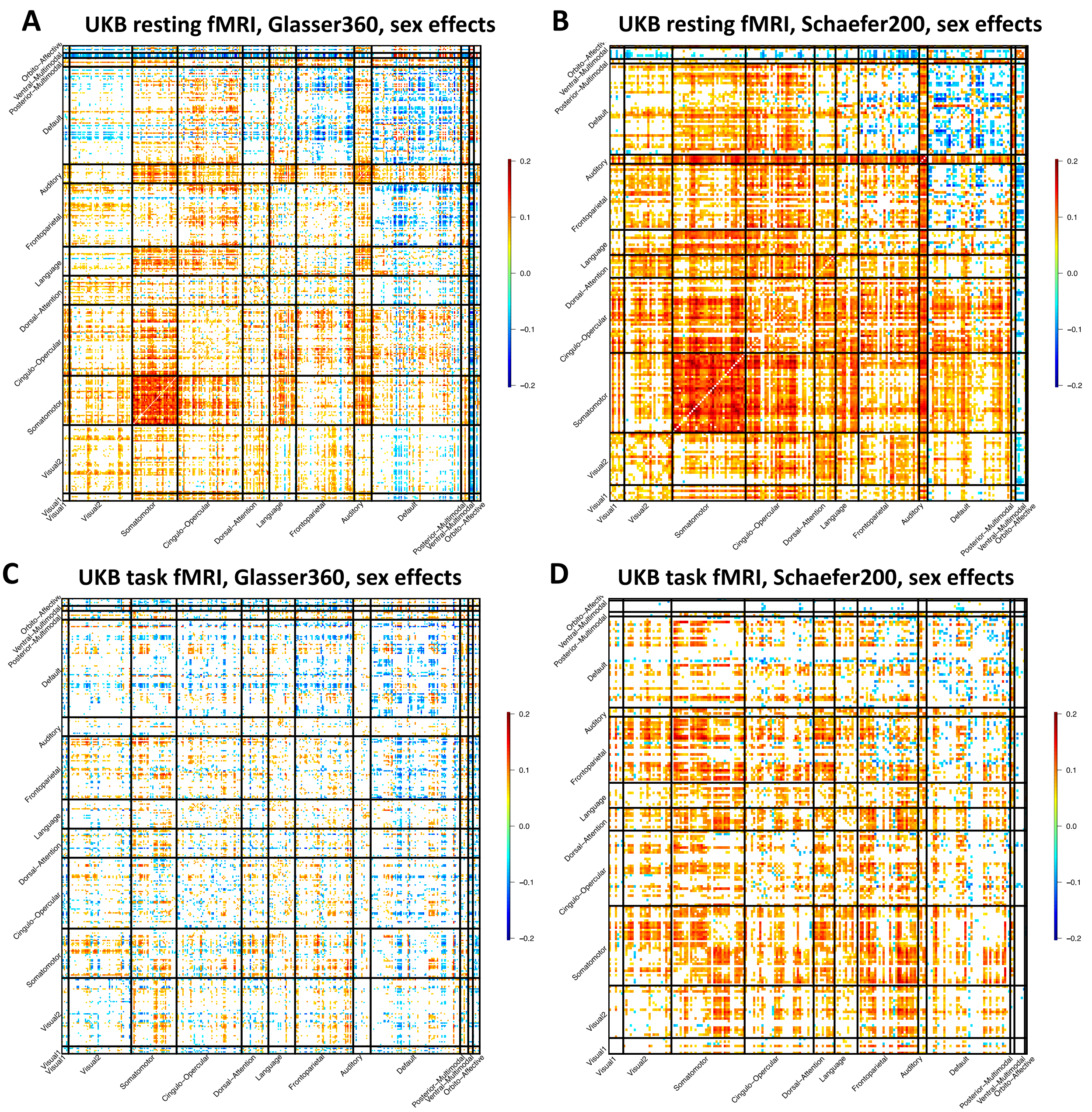


**Fig. S23 Comparison of the sex effect patterns in the Glasser360 and Schaefer200 atlases.**

We illustrated the effects passing the Bonferroni significance level (64,620 tests for Glasser360 and 19,900 tests for Schaefer200) in the discovery dataset (*n* = 33,795 for resting and 28, 907 for task) and also being significant at the nominal significance level (0.05) in the validation dataset (*n* = 5, 961 for resting and 4, 884 for task).

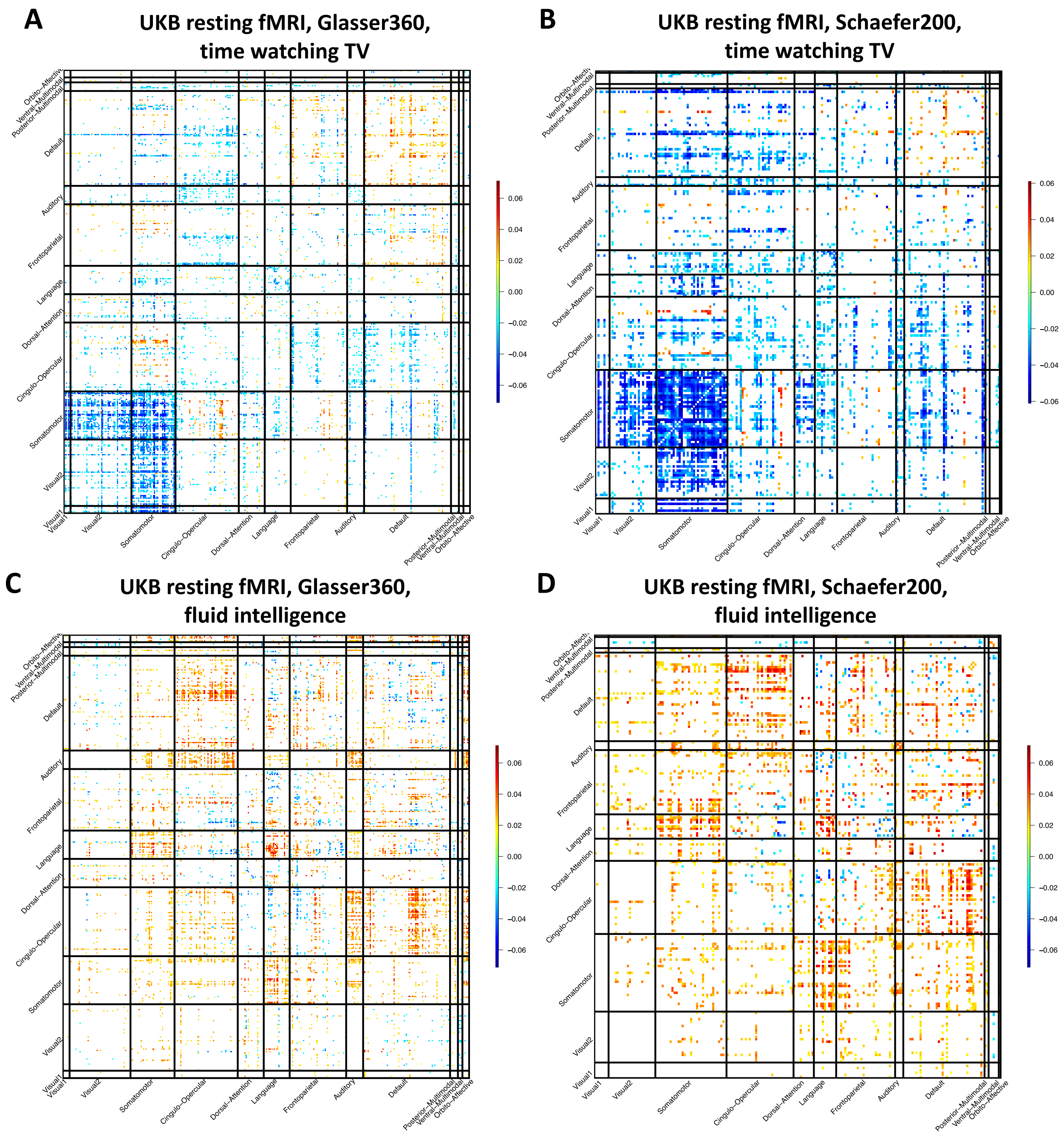


**Fig. S24 Comparison of association patterns in the Glasser360 and Schaefer200 atlases.**

We illustrated the correlation coefficients that were significant at FDR 5% level in the discovery dataset (n = 33,795) and were also significant at the nominal significance level (0.05) in the validation dataset (n = 5, 961). Time watching TV, time spent watching TV (Data field 1070); and fluid intelligence, fluid intelligence score (Data field 20127).

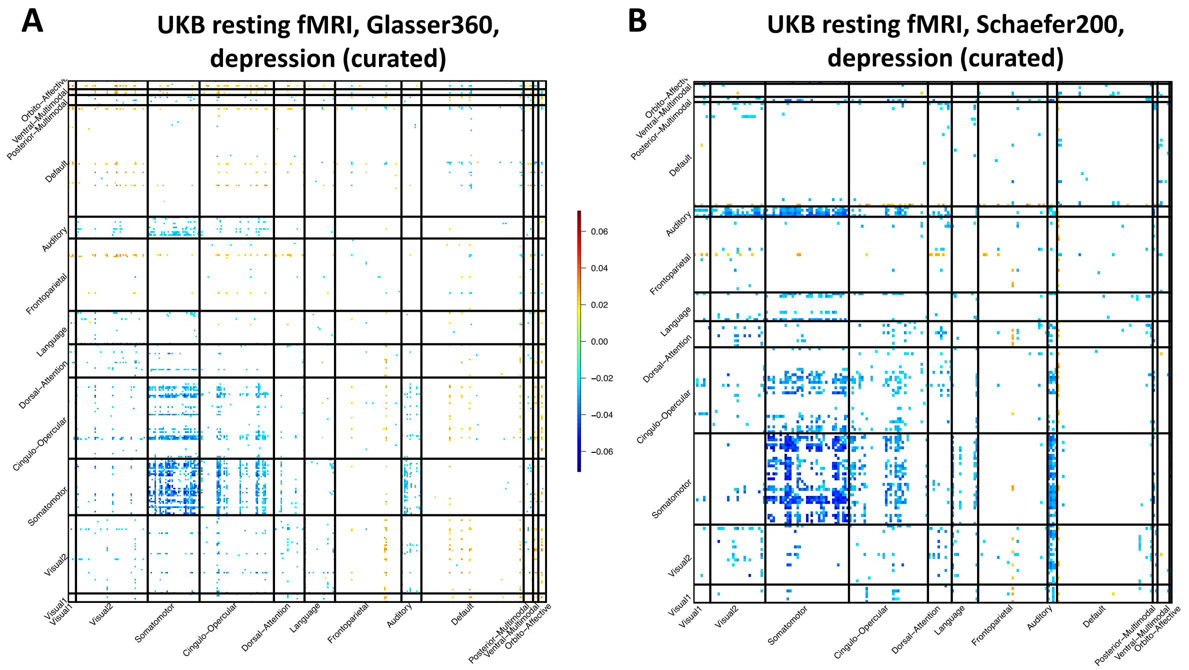


**Fig. S25 Comparison of association patterns in the Glasser360 and Schaefer200 atlases.**

We illustrated the correlation coefficients that were significant at FDR 5% level in the discovery dataset (n = 33,795) and were also significant at the nominal significance level (0.05) in the validation dataset (n = 5, 961). Time watching TV, time spent watching TV (Data field 1070); and fluid intelligence, fluid intelligence score (Data field 20127).

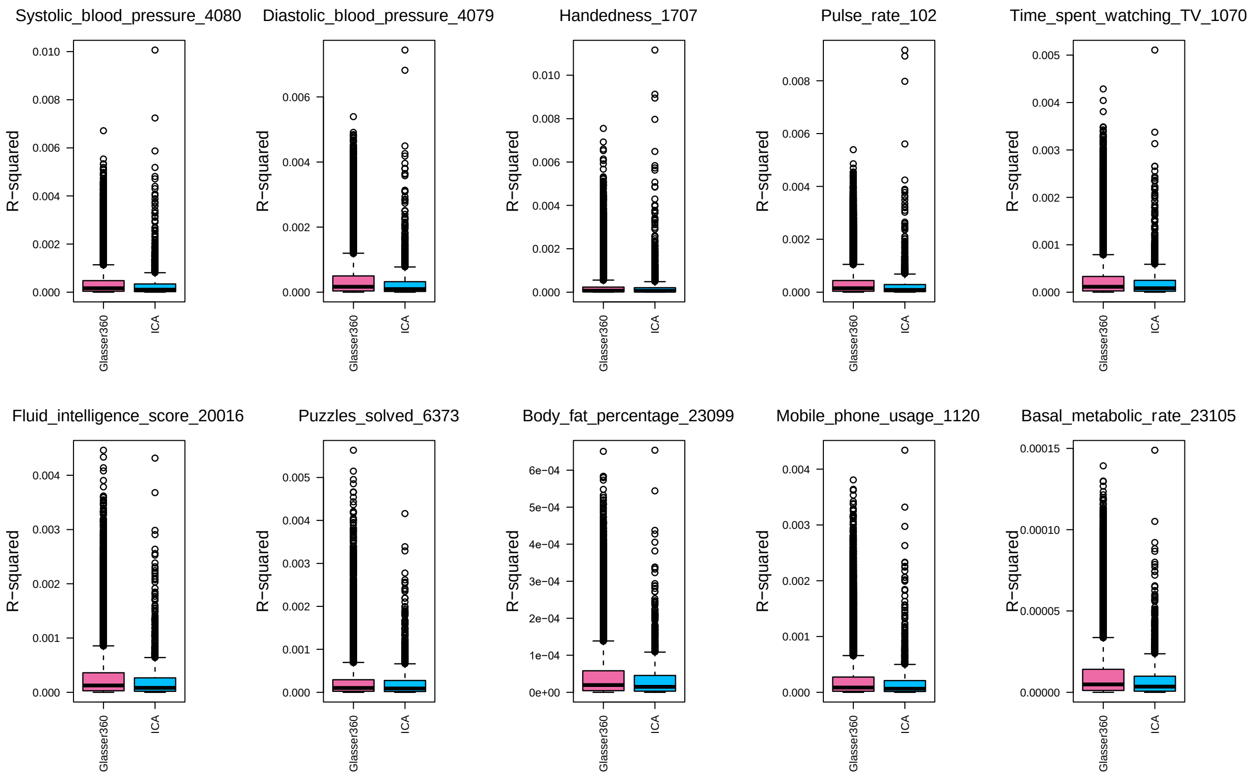


**Fig. S26 Comparison of selected trait associations with the Glasser360 atlas traits and whole brain ICA traits.**

We ranked all traits by their associations with the while brain ICA functional connectivity traits and plotted the top 10 ranked traits. In this figure, we illustrated the r-squared of the associations with the traits from the Glasser360 atlas traits (Glasser360) and whole brain ICA traits (ICA).

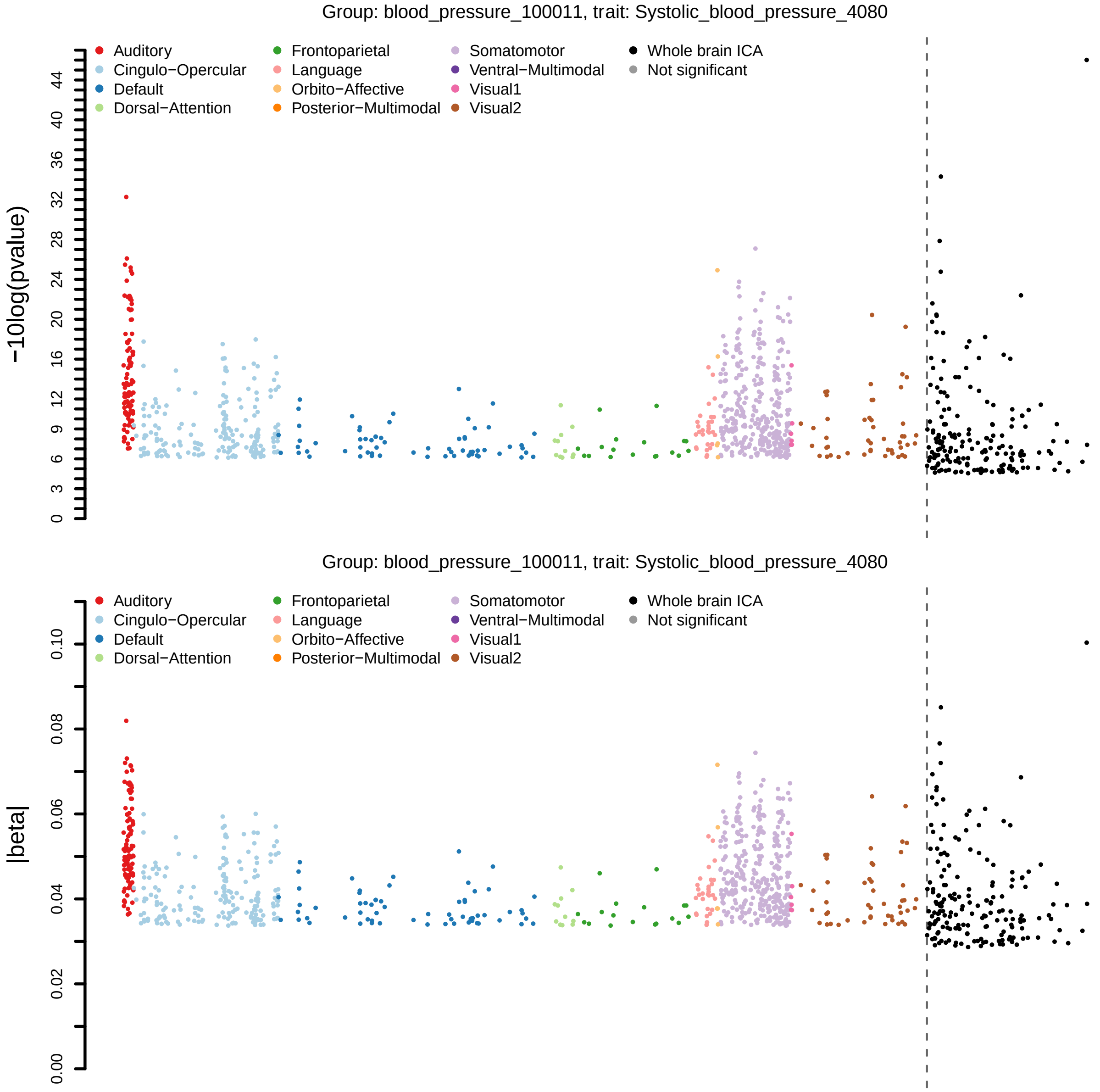


**Fig. S27 Comparison of systolic blood pressure's association with the Glasser360 atlas traits and whole brain ICA traits.**

We illustrated the -log10(pvalue) and regression coefficient (beta) of the associations with the traits from the Glasser360 atlas traits and whole brain ICA traits. Bonferroni-significant traits are highlighted with colors.
